# Stepwise Humanization of the Yeast TRAPP Core Enables Functional Analysis of TRAPP Variants

**DOI:** 10.3390/cells15171555

**Published:** 2026-08-27

**Authors:** Chelsea Abboud, Rozmehr Shokohi, Olivia Pape, Mahsa Mehranfar, Emma L. Baple, Nadirah Damseh, Michael Sacher

**Affiliations:** 1 Department of Biology, Concordia University, Montreal, QC H4B 1R6, Canada; 2 Department of Clinical and Biomedical Sciences, Faculty of Health and Life Sciences, University of Exeter, Exeter EX1 2LU, UK; 3 South West Clinical Genetics Service, Royal Devon University Healthcare NHS Foundation Trust, Exeter EX1 2LU, UK; 4 Department of Pediatrics and Genetics, Makassed Hospital, Al-Quds Medical School, Jerusalem 19482, Israel; 5 Department of Anatomy and Cell Biology, McGill University, Montreal, QC H3A 0C7, Canada

**Keywords:** TRAPP, variants of uncertain significance, humanization, yeast, genome editing

## Abstract

The Transport Protein Particle (TRAPP) complex is a highly conserved multi-subunit tethering complex that plays a critical role in membrane trafficking. Mutations in TRAPP complex subunits have been implicated in a growing spectrum of rare genetic disorders, yet the molecular mechanisms underlying variant pathogenicity often remain unclear. Here, we developed a humanized yeast platform to enable systematic functional characterization of TRAPP complex variants of uncertain significance. Using a stepwise gene replacement strategy in *Saccharomyces cerevisiae*, we constructed a strain in which five yeast TRAPP core subunits were replaced with their human orthologues. The integration of human subunits was validated through quantitative RT-PCR and Western blotting. Growth assays revealed that partial humanization of the core complex recapitulates key functional aspects of TRAPP assembly and enables the functional investigation of variants of uncertain significance in vivo. Structural modeling and clash analysis provided insights into the impact of specific mutations on complex stability and subunit interactions. TRAPPC3 has not yet been definitively associated with human disease. Introduction of TRAPPC3 variants of uncertain clinical significance into the humanized strain resulted in pronounced growth defects and predicted structural clashes. This work demonstrates the power of humanized yeast as a model for elucidating potential genotype–phenotype relationships in TRAPPopathy disorders and provides a versatile platform to support variant interpretation, mechanistic studies, and potential therapeutic screening.

## 1. Introduction

It is estimated that 300 million people worldwide are affected by some form of a rare disease [1]. Patients often endure a lifelong “diagnostic odyssey”, lasting an average of 6 years before receiving an accurate diagnosis. This delay often imposes profound impacts in terms of chronic, severe, and life-threatening symptoms in some patients. The delay is due to several reasons, including heterogeneity of symptoms, low clinical awareness, and limited research data for each condition. Additionally, since patient numbers are low and procedures to acquire patient cells are invasive, studying rare diseases in human populations is difficult and often only possible in model organisms such as yeast, worms, flies and fish [2]. Many human genes linked to rare disease have orthologues in model organisms, enabling the creation of disease-causing mutations in an experimental setting.

Yeast (*Saccharomyces cerevisiae*) is an invaluable tool for revealing human cellular pathways and disease [3]. Through the use of yeast, entire gene products have been assigned to genes solely through genetics, as has been done for many of the genes involved in secretion [4]. Further strengthening the use of this model system to study human proteins is the fact that human genes are widely conserved in yeast [5], opening up the possibility of humanization of a particular locus [6].

A classical use is to test for functional complementation, where one tests whether the human ORF can replace the function of the yeast gene [7]. This was demonstrated when the human *RAS* gene was expressed in yeast, proving to complement the yeast *RAS* gene in a *ras* knockout [8]. Since then, hundreds of human genes, with a focus on essential genes, have been successfully humanized in yeast. A seminal study that replaced 414 yeast genes with their human orthologues one by one demonstrated that 47% of the human genes (essential in yeast) could complement their yeast orthologue [6]. It is worth noting that humanization does not have to be limited to single genes. For example, the humanization of the glycolysis pathway in yeast has been possible through the replacement of 10 human enzymes in yeast [9]. This generated a yeast strain that was able to carry out glycolysis using the complete set of human enzymes; however, certain enzymes required adaptive mutations to achieve optimal functionality in the yeast cellular environment. There is evidence indicating that humanization of complexes is possible. One example is the human proteasome complex, which breaks down proteins in the cell that are no longer needed. Nearly all human proteasome subunits were replaceable [10]. In addition, humanization of the nucleosome was achieved by replacement of the four core histones H2A, H2B, H3 and H4 with their human counterparts. Viable humanized colonies arose only rarely and after prolonged selection, and subsequent acquisition of suppressor mutations improved cellular fitness [11].

In the context of rare diseases, patient-derived cells are often not readily available, making timely functional validation of variants of uncertain significance (VUS) particularly challenging. A humanized yeast model offers a powerful alternative for rapidly characterizing disease-causing variants. Combining emerging methods such as saturation mutagenesis and next-generation sequencing (NGS) with a humanized model organism enables high-throughput functional screening of large variant libraries. This approach provides a direct link between genotype and phenotype, supporting the interpretation of VUS in a clinically relevant timeframe.

**Figure 1:**
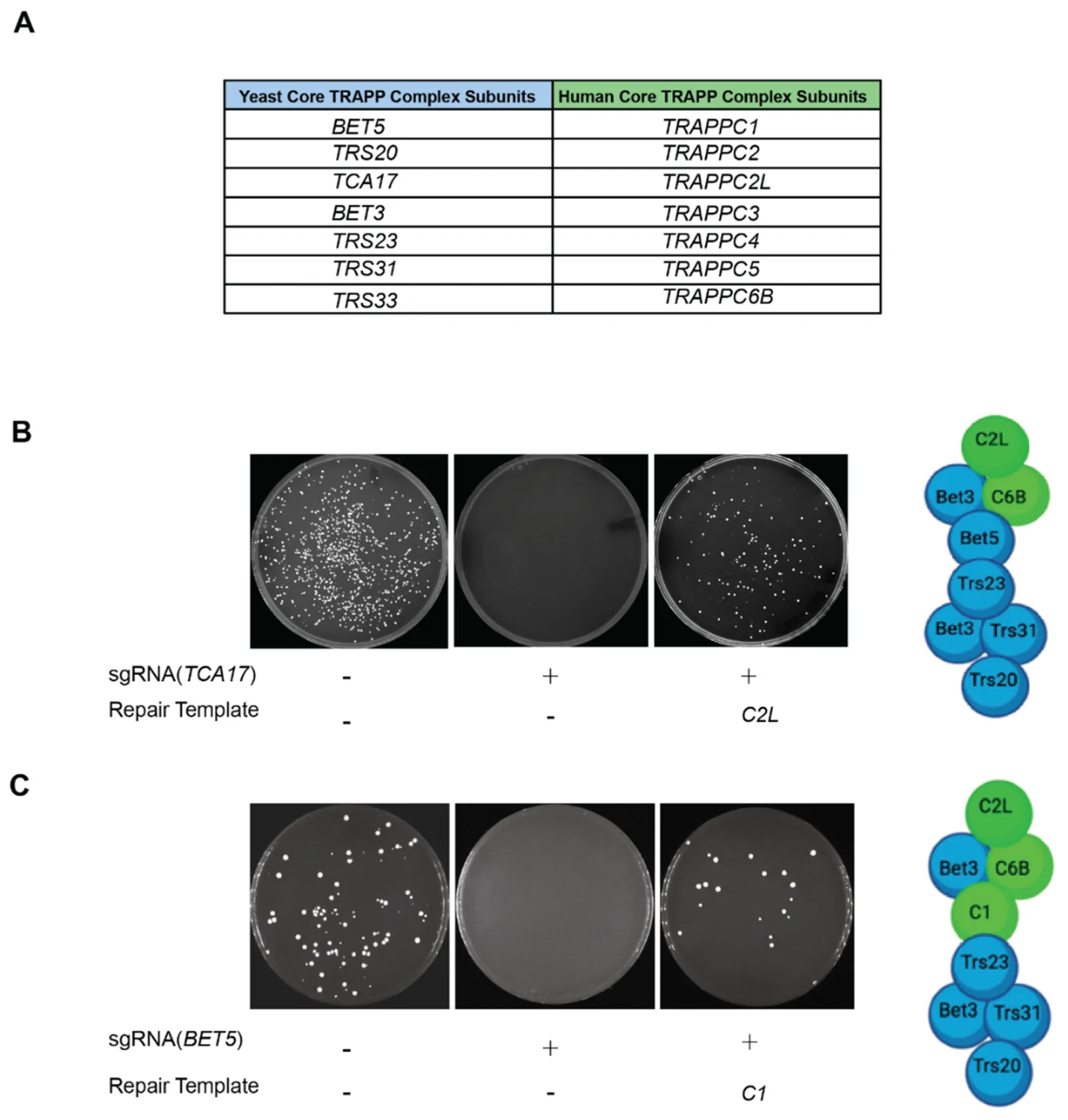
CRISPR-Cas9-mediated humanization of TRAPP complex subunits in yeast. (**A**) Schematic representation of the yeast TRAPP core showing the corresponding human orthologue for each subunit. (**B**) Replacement of *TCA17* with *C2L* in a C6B-humanized strain. (**C**) Replacement of *BET5* with *C1* in a C2L-C6B-humanized strain. The column with no sgRNA and no repair template (empty plasmid) serves as a control for demonstrating baseline transformation efficiency. sgRNA without repair template is a control to verify specificity of sgRNA; absence of growth confirms that sgRNA double-stranded breaks are lethal without repair. sgRNA with repair template shows successful integration of the human TRAPP subunit ORF, as indicated by colony growth. Transformants were selected on YPD+G418 medium to confirm successful integration.

The Transport Protein Particle (TRAPP) complex is a multi-subunit complex that is highly conserved and functions in intracellular vesicle trafficking. It was initially identified in yeast and shown to function in endoplasmic reticulum (ER)-to-Golgi transport [12]. Subsequent work in yeast identified three related complexes called TRAPPI, TRAPPII, and TRAPPIII [13,14]. All three share a common core of subunits but include distinct accessory subunits that tailor each complex to a specific pathway. More recently, yeast TRAPP I was suggested to be an in vitro artifact, with only TRAPP II and III having physiological relevance [15]. In addition, in vivo work suggests the presence of only TRAPP II and III [16,17]. The TRAPP core is composed of Bet3 (two copies), Bet5, Trs20, Trs23 Trs31, and [12]. Two TRAPP complexes (II and III) were also identified in humans [18,19]. Both of these complexes share a conserved common core of subunits (TRAPPC1, TRAPPC2, TRAPPC2L, TRAPPC3, TRAPPC4, TRAPPC5, and TRAPPC6A/B). The correspondence between yeast TRAPP core subunits and their human orthologues is shown in Figure 1A. In mammalian cells, the TRAPP complexes play distinct roles in regulating membrane trafficking pathways through specific interactions with Rab GTPases. TRAPP II has been implicated in the regulation of Rab11-dependent trafficking, and TRAPP III regulates early secretory pathway trafficking and autophagy through Rab1-dependent processes [19,20].

The structure of the yeast TRAPP core was elucidated by Kim et al. (2006) using a combination of crystallography and electron microscopy [21]. Their study revealed that the seven core subunits form a flat, elongated bi-lobed complex approximately 135 Å in length and 30–75 Å in thickness. This architecture arises through lateral juxtaposition of subunits, with two Bet3 molecules occupying central positions on each lobe, and Bet5 and Trs23 contributing to a symmetrical interface. Trs31 is located at one end of the complex, establishing contacts with Trs23 and Bet3 [21].

TRAPPopathies is the collective term for a growing set of rare disorders caused by mutations in genes encoding subunits of the TRAPP complexes [22]. TRAPPopathies usually fall into three major clinical categories: neurodevelopmental disorders, muscular dystrophies, or skeletal dysplasias that often manifest in early life [22,23]. Moreover, pathogenic variants have been identified in several subunits, and each can give rise to a distinct phenotype.

Previous work on the humanization of the TRAPP complex has shown that TRAPPC1, C2, C2L, and C6A/B could individually substitute for their yeast orthologues (note: for simplicity, the TRAPP portion of the protein names will be dropped hereafter) [24]. However, C3, C4, and C5 could not functionally substitute for their respective yeast orthologues. This was surprising, particularly for C3, which displays high similarity with its yeast counterpart. Importantly, work on C1-humanized yeast demonstrated that it is a valid model to characterize VUS [24]. This work provided a strong starting point but also highlighted the need for further investigation into the factors that limit humanization of some TRAPP subunits. We hypothesized that human TRAPP subunits that could not individually substitute for their yeast orthologues would require the presence of their closest human neighboring subunit in the complex for proper incorporation.

Here, we tested this hypothesis by rationally humanizing TRAPP in yeast based on the known architecture. We found that, indeed, genes that could not be individually humanized could in fact be humanized after prior humanization of the genes encoding neighboring proteins within the TRAPP core. We then used this new humanized yeast system to initially examine a TRAPPC3 VUS and show that it is indeed dysfunctional. This work demonstrates the power of model system humanization in human disease genetics and opens the door to rapidly evaluating all TRAPP core VUS.

## 2. Materials and Methods

### 2.1. Plasmids, Strains and Media

A list of all plasmids and yeast strains utilized in this study, including their relevant genetic backgrounds and construction details, is provided in Table 1 and Table 2.

Standard YPD medium (1% yeast extract, 2% peptone, 2% glucose) was used for routine yeast growth and propagation. For selection of yeast transformants carrying the KanMX cassette, YPD was supplemented with 200 µg/mL G418. Synthetic complete dropout media were used for plasmid selection. For bacterial culture, LB medium (1% tryptone, 0.5% yeast extract, 1% NaCl) was used to propagate *E. coli* strains carrying plasmids.

### 2.2. Humanization of Yeast

Humanization was performed using Frozen-EZ Yeast Transformation II Kit (Zymo Research, Irvine, CA, USA) according to the manufacturer’s protocol. A 60 min recovery in YPD was added prior to plating 250 µL of cells.

### 2.3. Standard Lithium Acetate Yeast Transformation

For non-CRISPR-CAS9 transformations, the standard lithium acetate/polyethylene glycol (PEG) method was used [26]. The transformed cells were resuspended in 300 µL of sterile water, and 150 µL of the suspension was plated onto selective plates to select for successful transformants.

### 2.4. Bacterial Transformation and Plasmid Propagation

Chemically competent *E. coli* cells from ThermoFisher (catalog number 18265017; Waltham, MA, USA) were used as per the manufacturer’s protocol.

### 2.5. Genomic DNA Extraction

Genomic DNA was extracted by first pelleting 1.5 mL of yeast culture at maximum speed for 1 min, followed by resuspension in 750 µL of sterile water. The cells were then resuspended in 500 µL of spheroplast medium, which consisted of 0.9 M sorbitol, 0.1 M EDTA, 30 mM β-mercaptoethanol, and 0.1–0.5 mg Zymolyase 100T. This mixture was incubated at 37 °C in a heat block. After incubation, the cells were pelleted again at maximum speed for 1 min and gently resuspended in 200 µL of Tris-EDTA buffer. To lyse the cells, 30 µL of 10% SDS was added, and the mixture was incubated at 70 °C for 15–30 min. Protein precipitation was achieved by adding 80 µL of 5 M potassium acetate, followed by incubation on ice for 30 min to 1 h. The lysate was centrifuged for 10 min, and the supernatant was transferred to a new tube and centrifuged again for another 10 min to further clarify the solution. DNA was then precipitated by adding an equal volume of isopropanol to the supernatant and centrifuging for 1 min. The resulting DNA pellet was washed with 500 µL of 70% ethanol and centrifuged for 30 s. Finally, the pellet was air-dried at 37 °C for 15 min and resuspended in 100 µL of 10 mM Tris/0.5 mM EDTA.

### 2.6. Confirmation PCR

Confirmation of humanization was achieved by PCR. The PCR primers were designed such that the forward primer anneals to the 5’ yeast untranslated region (UTR) and the reverse primer anneals to the human ORF of interest. The primers used are listed in Table 3. PCR reactions were performed and analyzed on a 1.5% agarose gel.

### 2.7. Sequencing of Humanized Genes

As a final confirmation step, the ORFs were amplified and sequenced. The primers used for PCR sequencing anneal to the regions flanking the 5′ and 3′ untranslated regions (UTRs), allowing amplification of the entire coding sequence including both UTRs (see Table 4).

### 2.8. sgRNA Design and Validation

Most of the sgRNAs were designed using Geneious (version 2025.2) and the Doench algorithm for high on-target/low off-target activity, with the exception of the *C4* sgRNA, which was designed using tools on the IDT website. BLAST v2.17.0 was used to predict potential off-target gRNA binding sites. The oligonucleotides used to generate the sgRNA to be cloned are listed in Table 5.

### 2.9. Site-Directed Mutagenesis

Site-directed mutagenesis was performed using Pfu-HF and mutagenic oligonucleotides (Table 6) for 18 cycles. Following thermocycling, the wild-type DNA template was digested with DpnI, and 2 μL of the reaction was transformed into bacteria by electroporation. Transformed cells were selected on LB+ampicillin plates, and mutagenesis was verified by sequence analysis.

### 2.10. Golden Gate Assembly for sgRNA Cloning

The sgRNA was cloned into bacteria using the Golden Gate assembly method. To facilitate cloning with the BsmBI-v2 enzyme, specific overhangs were added to the oligonucleotides. For the forward oligo, GACTTT was added at the 5′ end, followed by the gRNA sequence. For the reverse oligo, AAAC was added at the 5′ end, followed by the reverse complement of the gRNA sequence, and ended with AA to complete the overhang for ligation. To prepare the sgRNA inserts for Golden Gate cloning, complementary forward and reverse oligos were annealed prior to ligation. For annealing, 2 µL of each oligo was mixed with 16 µL of nuclease-free water to reach a final concentration of 10 µM in a total volume of 20 µL. The annealing reaction was performed in a thermal cycler using the following conditions: 95 °C for 15 min, decreasing the temperature by 5 °C every 15 min until 25 °C was reached. The annealed oligos were then diluted 1:1000, and 2 µL of the dilution was used for the Golden Gate assembly. The vector used was pCEN6-Cas9-GFP-KanX (kind gift from Dr. A. Kachroo). The reaction was performed in a 10 µL volume and contained the following components: 20 fmol of circular pCEN6-Cas9-KanMX-GFP vector, 20 fmol of annealed sgRNA oligos, 1 µL of 10× r3.1 buffer (NEB), 1 µL of BsmBI-v2 restriction enzyme, 1 µL of 10 mM ATP, and 1 µL of T7 DNA ligase. The reaction mixture was subjected to thermal cycling to enable efficient digestion and ligation. The program consisted of alternating incubations at 37 °C for 5 min (to facilitate BsmBI digestion) and 16 °C for 5 min (to promote ligation), repeated for 30 cycles. This was followed by a final incubation at 37 °C for 60 min and an enzyme inactivation step at 85 °C for 15 min. Subsequently, 5 µL of the Golden Gate reaction was transformed into 50 µL of chemically competent *E. coli* cells. Transformants were plated on LB agar supplemented with kanamycin, and green-white screening was used to identify colonies that successfully incorporated the sgRNA insert in place of GFP. In successful transformants, the GFP cassette is excised and replaced by the sgRNA insert, resulting in non-fluorescent colonies.

### 2.11. Liquid Growth Curve Assays 

Growth curves were conducted using a microplate reader at three different temperatures. At 25 °C, absorbance measurements were taken using a Tecan Sunrise microplate reader (Männedorf, Switzerland), while growth at 30 °C and 37 °C was monitored using an Agilent BioTek LogPhase 600 microplate reader (Winooski, VT, USA). Yeast cultures were diluted to an initial optical density (OD_600_) of 0.01. To minimize edge effects and evaporation, the peripheral wells of the 96-well plate were filled with phosphate-buffered saline (PBS). Additionally, a Breath Easy sealing film (Sigma) was applied to the plate. Growth was continuously monitored over a period of 48 h. All growth curve experiments were performed in triplicate with at least three independent biological replicates, and data are presented as the mean of all replicates.

### 2.12. Spot Dilution Assays 

Yeast cultures were adjusted to an OD_600_ of 0.5, and three successive 1:10 serial dilutions were prepared. From each dilution, 2 µL was spotted onto the appropriate solid media. Plates were incubated at the designated temperature (25 °C, 30 °C, 37 °C), and growth was monitored. Images were taken after 24 and 48 h to assess growth differences across strains and conditions. This experiment was repeated at least three times.

### 2.13. Western Analysis

Western blotting was used qualitatively to assess the presence or absence of the indicated proteins and was not used for quantitative comparison of protein abundance between strains. The lysates for Western blots were prepared in two ways: the spheroplast method and the glass beads method. For the spheroplast method, 10 OD units of cells were pelleted by centrifugation at maximum speed for 1 min in a tabletop centrifuge. The pellet was washed with 750 µL of sterile water, then resuspended in 1 mL of spheroplasting buffer (see Genomic DNA Isolation for recipe) and incubated for 1 h at 37 °C in a heat block. After incubation, cells were pelleted again at maximum speed for 1 min and washed with 750 µL of spheroplasting buffer without zymolyase. The final pellet was resuspended in 100 µL of 10 mM Tris, 0.5 mM EDTA, 1% SDS. Protein concentration was determined using the Bradford assay reagent (BioRad, Hercules, CA, USA).

For the glass beads method, 2 OD units of cells were harvested and the supernatant discarded. The pellet was resuspended in 1 × SDS-PAGE sample buffer at a volume of 20–30 µL per OD unit. An equal volume of acid-washed glass beads was added to each tube. The samples were vortexed for 2 min, then boiled for 2 min. This cycle was repeated a total of three times. Tubes were centrifuged at maximum speed in a microfuge for 1 min, and the supernatant was transferred to a fresh microcentrifuge tube. Equal volumes of lysate were loaded onto the SDS-PAGE gel.

### 2.14. Real-Time RT-PCR

Total RNA was extracted from yeast samples using the YeaStar™ RNA Kit (Zymo Research, Irvine, CA, USA) following the manufacturer’s protocol. Real-time PCR conditions were performed using subunit-specific primers, listed in Table 7**.** For reactions involving the C1 subunit, the reverse primer was used at a concentration of 150 nM instead of the standard 100 nM. Real-time RT-PCR was used in this study to determine whether transcripts from the introduced human genes were detectable in the humanized strains. Because expression was not normalized to an internal reference gene, these experiments were not used for quantitative comparison of transcript abundance between strains.

### 2.15. In Silico Structural Analyses

#### 2.15.1. Starting Structures

The yeast TRAPP core was derived from the cryo-EM structure of the yeast TRAPPII complex (PDB 7E2D [27]); TRAPPII-specific subunits were removed in silico to leave the seven core chains—Tca17 (chain A), Trs33 (chain B), Bet3 (chains C and F), Bet5 (chain D), Trs23 (chain E), Trs31 (chain G), and Trs20 (chain H). Human subunit models were obtained from the AlphaFold Protein Structure Database (AlphaFold monomer v2.0 predictions): TRAPPC1 (UniProt Q9Y5R8), TRAPPC2L (Q9UL33), TRAPPC3 (O43617), TRAPPC4 (Q9Y296), and TRAPPC6B (Q86SZ2). For the C4ΔPDZL construct, the PDZ-like domain was removed from the TRAPPC4 model by deleting residues 26–100, retaining residues 1–25 and 101–219.

#### 2.15.2. Positioning of Human Subunits

Each human subunit model was superposed onto its yeast counterpart within the 7E2D core using the ChimeraX MatchMaker (version 1.10) tool with default settings, after which the yeast subunit was deleted, leaving the human subunit positioned in the complex for clash detection against the surrounding subunits. Models were analyzed as predicted and superposed; no energy minimization or geometry optimization was performed.

#### 2.15.3. Clash Analysis

Steric clashes were identified with the Clashes tool in UCSF ChimeraX version 1.10 (2025-06-26). An atom pair was flagged as a clash when the van der Waals overlap was ≥ 0.60 Å after subtracting a 0.40 Å allowance for hydrogen bonding; atom pairs separated by four or fewer bonds were ignored. Both intermodel and intramodel clashes were included and rendered as red pseudobonds (0.15 Å radius, four dashes per bond). The two clash figures differed only in the sequence-separation exclusion. For the C4ΔPDZL versus C4 comparison (Figure 5), no sequence-separation exclusion was applied so that the full set of overlaps introduced by the intact PDZL domain would be captured and all clashes among the displayed subunits, including those between residues five or fewer apart in sequence, are shown. For the sequential-humanization series (Figure 7), clashes between residues fewer than five apart in sequence were excluded.

#### 2.15.4. TRAPPC3 Mutant Analysis

Structural analysis of TRAPPC3 variants (Figure 10) was performed using the PyMOL Molecular Graphics System, version 3.1.3.1 (Schrödinger, LLC, New York, NY, USA). The experimentally determined crystal structure of human Bet3/TRAPPC3 (PDB ID: 1SZ7) was used as the starting structural model. The missense variants p.R62W, p.R164W, p.L131F, and p.I51T were introduced individually using the PyMOL Mutagenesis Wizard. For each substitution, the available side chain rotamers generated by PyMOL were examined, and the rotamer with the lowest apparent steric interference with the surrounding structure was selected based on the clash assessment provided by the Mutagenesis Wizard. Residues containing atoms within 5 Å of the substituted residue were then identified to define the local structural environment. This region was visually inspected for changes in side-chain packing and for potential steric contacts or clashes introduced by the amino-acid substitution. Neighboring residues involved in potential steric contacts or altered local packing were highlighted in the structures. No molecular-dynamics simulation or energy-minimization analysis was performed following mutagenesis.

## 3. Results

### 3.1. Humanization and Validation of the Replacement TRAPP Core Subunits C1, C2L, and C6B

Previous work from our laboratory established that humanized yeast serves as an efficient system to study TRAPP variants of uncertain significance (VUS) [24]. The CRISPR-based humanization strategy involved introducing a plasmid encoding Cas9 and an sgRNA targeting the yeast orthologue. This system induces a double-stranded break (DSB) in the yeast genome, which is repaired using a human ORF as the repair template. The plasmid also confers G418 resistance for selection on YPD+G418 plates. After PCR validation and sequencing confirmation of correct integration, strains were streaked on YPD to eliminate the plasmid and G418 resistance, ensuring scarless replacement of the yeast gene with its human counterpart while maintaining an otherwise fully yeast background.

Previous work showed that the yeast genes encoding the orthologues of C1, C2, C2L, and C6B could each be individually replaced by their corresponding human genes, whereas replacement with C3, C4, or C5 was unsuccessful [24]. We hypothesized that the inability to replace some yeast TRAPP subunits with their human orthologues might reflect a requirement for neighboring subunits to also be humanized. Therefore, we began constructing a stepwise humanized TRAPP core in yeast, starting with a strain containing C6B, followed by sequential addition of C2L and C1 (Figure 1A,B). Although each of these human proteins can individually functionally substitute for its yeast orthologue, this approach allowed us to build a partially humanized TRAPP core in a yeast background. PCR followed by Sanger sequencing verified the scarless integration of these three human subunits (Figure S1).

**Figure 2:**
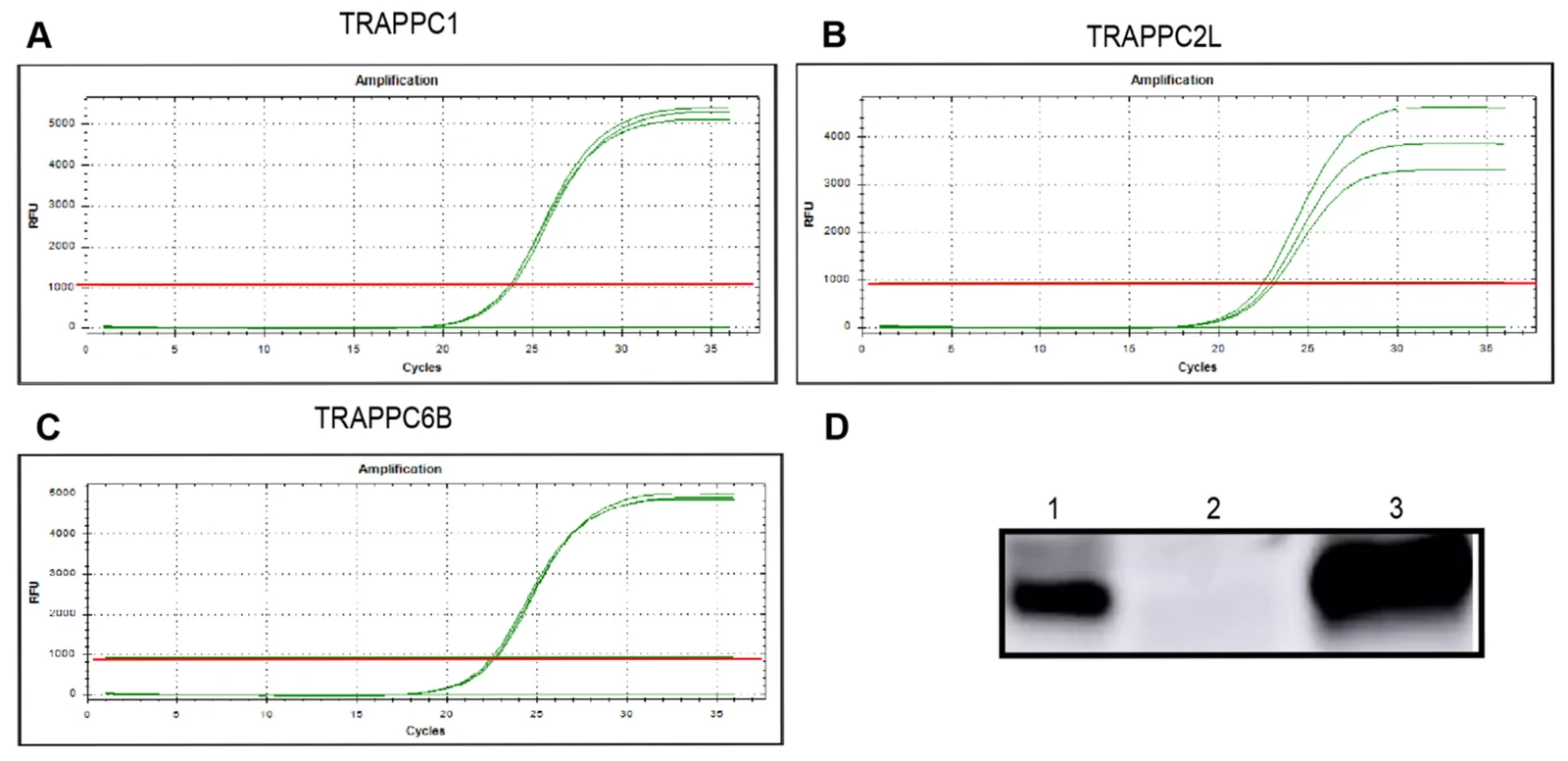
Real-time RT-PCR detection of human *TRAPPC1*, *TRAPPC2L*, and *TRAPPC6B* transcripts in humanized yeast strains. (**A**) Real-time RT-PCR amplification curves for *TRAPPC1*, (**B**) *TRAPPC2L*, and (**C**) *TRAPPC6B*, each showing three biological replicates as distinct peaks. In all panels, the red flat line at the baseline represents results for the parental yeast strain, in which no amplification was detected. An amplification threshold of 1000 RFU (relative fluorescence units) was applied, and only samples containing the corresponding human transcripts exceeded this value. (**D**) Yeast lysates were prepared from a non-humanized strain (lane 1), a strain in which C6B replaced Trs33 (lane 2) and a Trs33 overproducer strain (lane 3). Western analysis using an anti-Trs33 antibody was performed as a qualitative validation of the presence or absence of Trs33 and was not used for quantitative comparison of protein abundance.

Due to the lack of suitable antibodies for many of the human TRAPP proteins, real-time RT-PCR was used to confirm the presence of transcripts from the human *C1*, *C2L* and *C6B* genes in the humanized strains. Amplification was detected for each human transcript in the corresponding humanized strain, whereas no amplification was detected in the parental strain, consistent with expression of the introduced human genes (Figure 2A–C). These experiments were used qualitatively and were not intended to compare transcript abundance between strains.

As additional qualitative protein-level validation, Western analysis was performed using an antibody against the yeast protein Trs33. As expected, the parental strain displayed a Trs33 immunoreactive band, whereas the humanized strain did not, consistent with replacement of the yeast *TRS33* (Figure 2D). Collectively, these results confirm successful integration of the human TRAPP genes, while RT-PCR confirms the presence of the corresponding human transcripts.

### 3.2. Replacement of Yeast TRAPP Subunits with C4 and C3 and Unsuccessful Restoration of the C4 PDZL Domain

**Figure 3:**
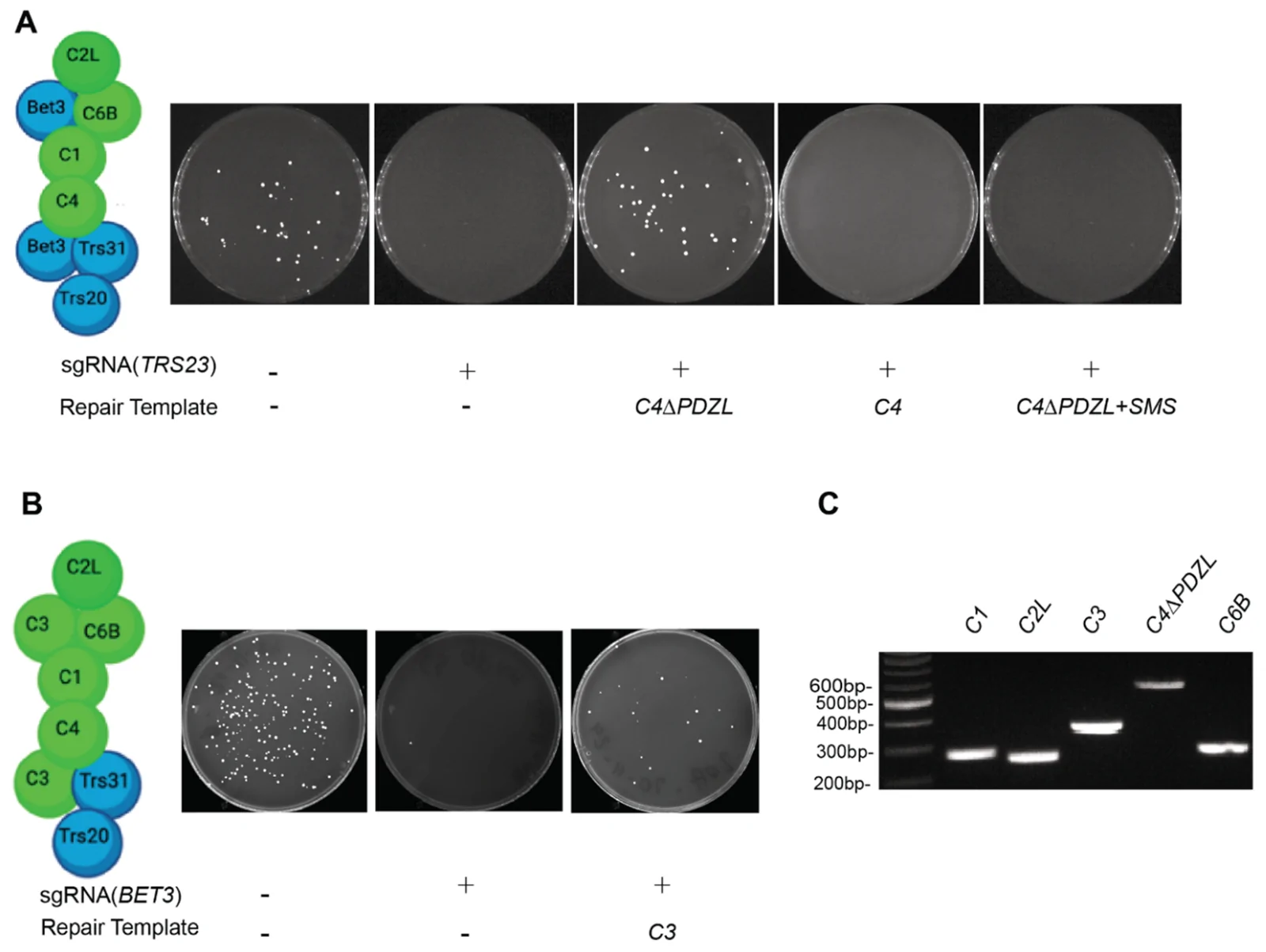
Successful sequential humanization of *C4∆PDZL* and C3. (**A**) Yeast transformation plates showing the outcome of attempts to repair the *TRS23* double-strand break with various repair templates: *C4∆PDZL*, *C4*, and *C4∆PDZL+SMS*. Plates contain YPD with G418 for selection. The presence of colonies indicates successful CRISPR-mediated replacement of the yeast gene with the human construct in the presence of both sgRNA (targeting *TRS23*) and the appropriate repair template. (**B**) Yeast transformation plates showing the CRISPR-Cas9-mediated replacement of *BET3* with human *C3*. Colonies on YPD+G418 selection plates are observed only when both the sgRNA (targeting *BET3*) and the *C3* repair template are present, indicating successful integration of the human *C3* sequence. Note that the two control plates (empty plasmid and sgRNA only) were part of the larger analysis of *C3* variants presented in Figure 9A and are reproduced there for completeness. (**C**) Confirmation PCR shows the successful integration of the human ORFs. PCR was performed using specific confirmation primers for each locus. The expected band size for each successful humanization is 274 bp for C1, 255 bp for C2L, 361 bp for C3, 562 C4∆PDZL, 276 bp for C6B.

To further humanize the TRAPP complex, we next decided to add the ORF encoding C4, which interacts directly with C1 [28]. Initial attempts to replace yeast *TRS23* with full-length human C4 were unsuccessful (Figure 3A). Drawing inspiration from previous work showing that making human proteins more yeast-like can enhance compatibility [10], we examined the structural differences between C4 and Trs23. We previously identified a PDZ-like (PDZL) domain in human C4 that is absent from the yeast orthologue, which instead contains a *Saccharomycotina*-specific (SMS) domain [15]. A truncated version lacking the PDZL domain (C4∆PDZL) was therefore generated as an engineered yeast-compatible derivative to test whether removal of this human-specific domain would permit functional substitution for yeast Trs23. This construct was able to successfully replace the yeast gene (Figure 3A). However, when further modified by adding the SMS domain from yeast Trs23, the replacement was unsuccessful. These results demonstrate that targeted domain removal can enable functional substitution by C4, but that additional modifications do not always improve compatibility and may actually hinder successful functional integration.

After establishing that C4ΔPDZL could successfully substitute for Trs23, we next attempted to replace *BET3* with *C3* in this partially humanized background. Importantly, previous attempts to replace *BET3* with *C3* in an otherwise yeast background were unsuccessful. However, when *C3* was introduced into the background already containing C4ΔPDZL, C1, C2, and C6B, successful replacement was achieved (Figure 3B). This finding demonstrates that prior replacement of certain neighboring yeast subunits with their human orthologues, particularly replacement of *TRS23* with *C4ΔPDZL*, is required to enable subsequent replacement of *BET3* with *C3*, highlighting the importance of context and compatible protein–protein interactions within the assembly of the TRAPP complex. The resulting strain, containing *C2L*, *C6B*, *C1*, *C4ΔPDZL*, *C3*, *TRS31*, and *TRS20*, is hereafter referred to as the Partially Humanized TRAPP Core (PHTC; MSY1061).

**Figure 4:**
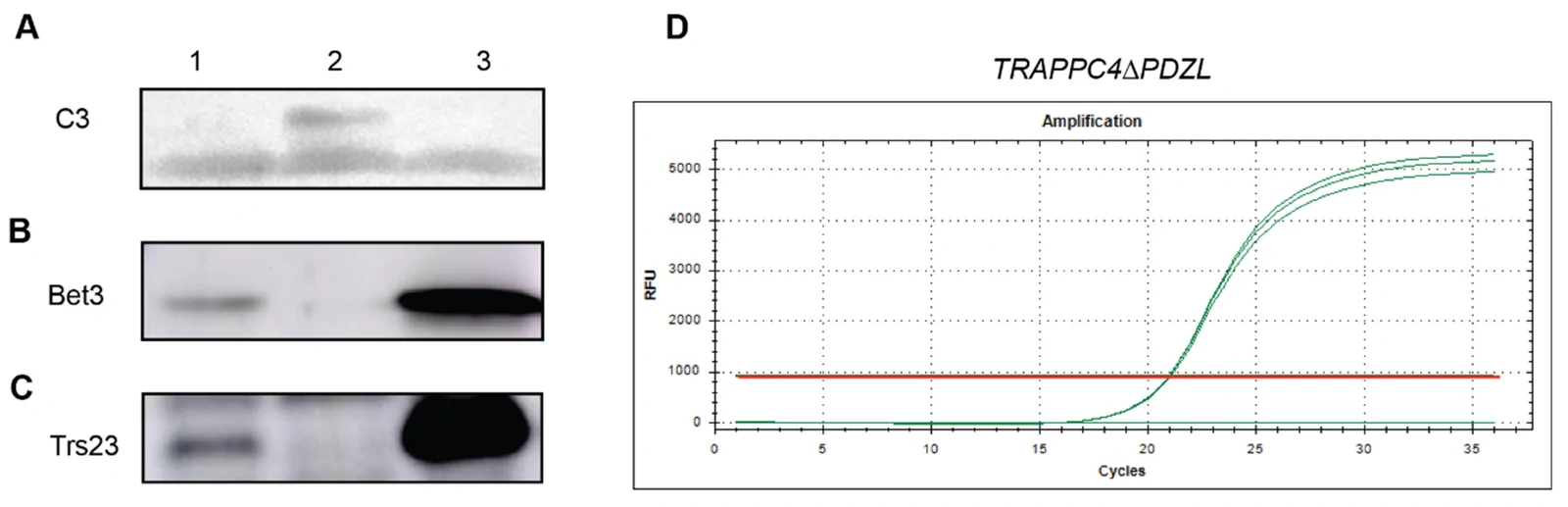
Confirmation of replacement of BET3 and TRS23 with their human orthologues. Yeast lysates were prepared from a non-humanized strain (lane 1), the PHTC strain (lane 2), and either a Bet3 overproducer strain (lane 3, panels (**A**,**B**)) or a Trs23 overproducer strain (lane 3, panel (**C**)). Lysates were analyzed by Western analysis using an anti-C3 antibody (panel (**A**)), an anti-Bet3 antibody (panel (**B**)), or an anti-Trs23 antibody (panel (**C**)). Western analyses in panels (**A**–**C**) were used qualitatively to assess the presence or absence of the indicated proteins and were not used for quantitative comparison of protein abundance between strains. (**D**) Real-time RT-PCR amplification curves for C4ΔPDZL showing three biological replicates as distinct peaks. The red flat line at the baseline represents the result for the parental yeast strain, in which no amplification was detected. An amplification threshold of 1000 RFU (relative fluorescence units) was applied, and only samples containing the C4ΔPDZL transcript exceeded this value.

To verify successful replacement, PCR analysis was performed for both C3 and C4ΔPDZL and demonstrated integration of the human genes at the expected loci (Figure 3C). Scarless gene replacement was further validated by Sanger sequencing, confirming precise substitution of the yeast genes with their human counterparts (Figure S2). The expression was then assessed by Western analysis for both humanized subunits and qRT-PCR for *C4ΔPDZL*. Western analysis using an antibody against human C3 detected a band in the humanized strain but not in the parental strain, providing qualitative evidence for expression of human C3 (Figure 4A). Conversely, an anti-Bet3 antibody detected Bet3 in the parental strain but not in the humanized strain (Figure 4B). Similarly, an anti-Trs23 antibody detected Trs23 in the parental strain but not in the strain in which *TRS23* was replaced by C4ΔPDZL (Figure 4C). The presence of the human *C4ΔPDZL* transcript was confirmed by real-time RT-PCR, with amplification detected in the humanized strain but not in the parental strain (Figure 4D), providing additional qualitative evidence for expression of the introduced human gene. Together, these findings demonstrate that both *C3* and *C4ΔPDZL* can functionally replace their essential yeast orthologues. This result underscores the importance of context-dependent humanization, where maintaining compatibility between protein–protein interactions within the complex is critical for successful assembly and function. Achieving functional expression of these essential subunits in yeast not only advances the construction of a fully humanized TRAPP core but also establishes a valuable platform for studying human variants in a tractable eukaryotic model.

**Figure 5:**
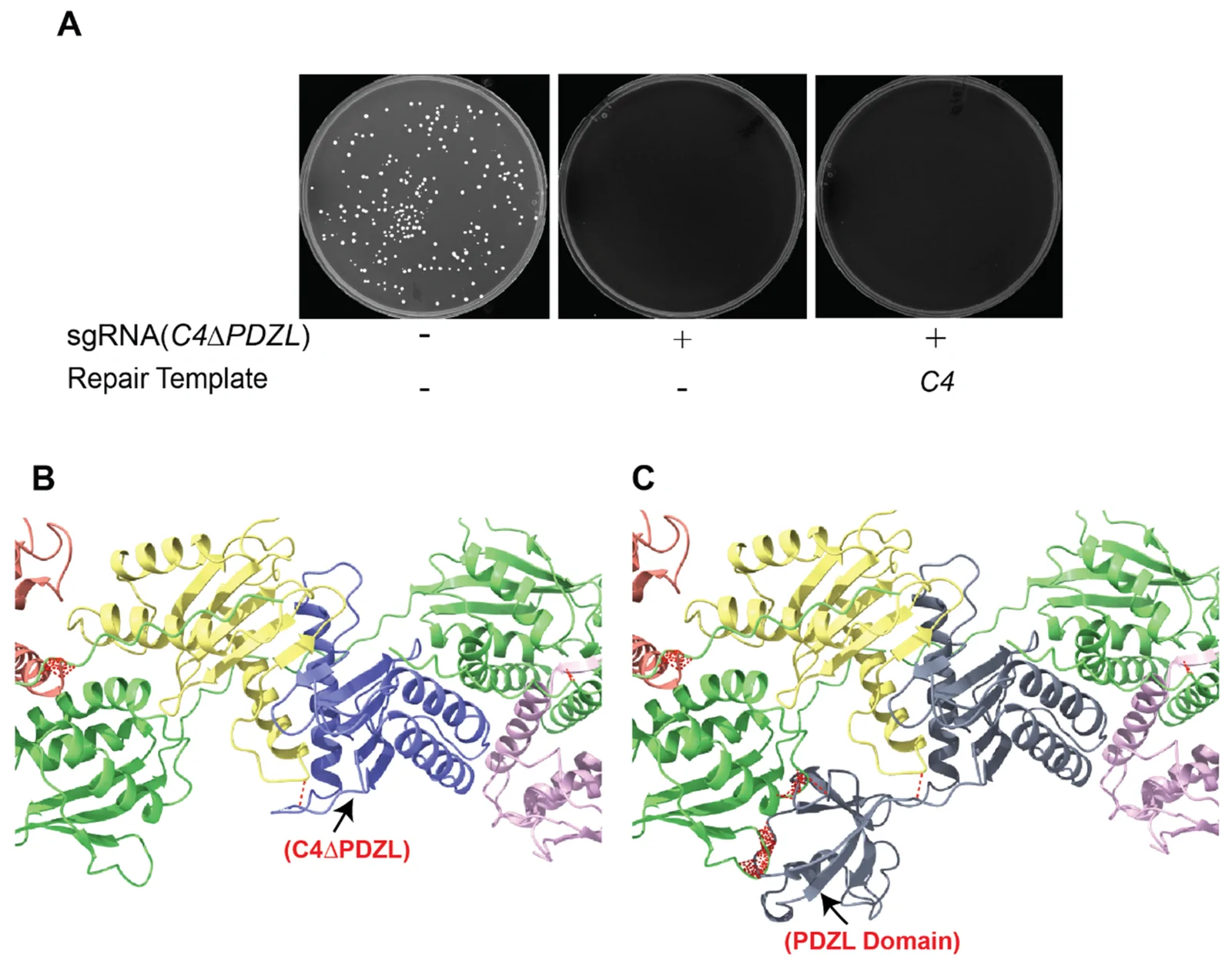
Unsuccessful replacement of *C4ΔPDZL* with *C4*. (**A**) CRISPR-Cas9 editing was performed in the PHTC strain. An sgRNA was designed to target the integrated human *C4∆PDZL* sequence, and the repair template used encoded the full-length human *C4* open reading frame (ORF). No colonies are observed in the plate that contains the sgRNA with the repair template, indicating unsuccessful replacement. (**B**) Structure showing the C3 (green), C1 (yellow), and C4ΔPDZL (purple) subunits. Dashed red lines indicate steric clashes detected using the ChimeraX built-in clash analysis tool with the following parameters: VDW overlap ≥ 0.60 Å (after subtracting 0.40 Å for H-bonding), center-to-center distance ≤ 4.0 Å, with at least one end selected. Interactions between atoms ≤ 4 bonds apart were ignored, but no interactions were excluded based on sequence separation (i.e., residues ≤ 5 apart in sequence were included). Only intermodel and intramolecular interactions were included. Pseudobonds representing clashes are displayed in red. (**C**) Same as (**B**), except the C4 subunit with intact PDZL domain is shown in nickel color.

After successfully humanizing *C3* in the context of the PHTC strain, we next attempted to restore the *PDZL* domain in *C4*, hypothesizing that the presence of humanized *C3* might enable successful replacement with the full-length human *C4*. Because *C4ΔPDZL* was already present in the PHTC strain, a new sgRNA was designed to specifically target the *C4ΔPDZL* allele. The full-length *C4* open reading frame was provided as a repair template for homologous recombination. However, this attempt to restore the *PDZL* domain was unsuccessful, as indicated by the absence of colonies on plates containing both the new sgRNA and the repair template (Figure 5A). To investigate the structural basis for the lack of viability, we performed clash analysis using ChimeraX, which shows any van der Waals overlap. In the PHTC context, a clash appears between *C1* and *C4∆PDZL* (Figure 5B). This clash is not lethal since *C4∆PDZL* can still replace *TRS23* in yeast. However, whenever *C4* is replaced in PHTC, new and more pronounced clashes with *C3* appear (Figure 5C). These results suggest that even in the presence of humanized *C3*, the addition of the *PDZL* domain is not tolerated in yeast, further highlighting the challenge of integrating human-specific domains into the TRAPP core complex.

*C2* and *C5* cannot replace their orthologues *TRS20* and *TRS31*, respectively, in the PHTC strain. Given that we constructed a strain (PHTC) that contains all human TRAPP core subunits except for *C5* and *C2*, we next attempted to complete the TRAPP core by adding the ORFs encoding these subunits. Attempts to replace yeast *TRS31* with human *C5* were unsuccessful, as evidenced by the lack of viable colonies following transformation on G418 plates (Figure S3). Additionally, efforts to humanize *C2* in the PHTC background did not result in successful replacement of *TRS20* (Figure S3). This result was unexpected, as human *C2* was previously shown to successfully replace *TRS20* in the parental yeast strain but failed to do so in the context of the PHTC background [24]. These findings suggest that the ability of certain human subunits to functionally replace their yeast counterparts may be highly dependent on the broader context of the complex and the specific combination of humanized subunits present.

**Figure 6:**
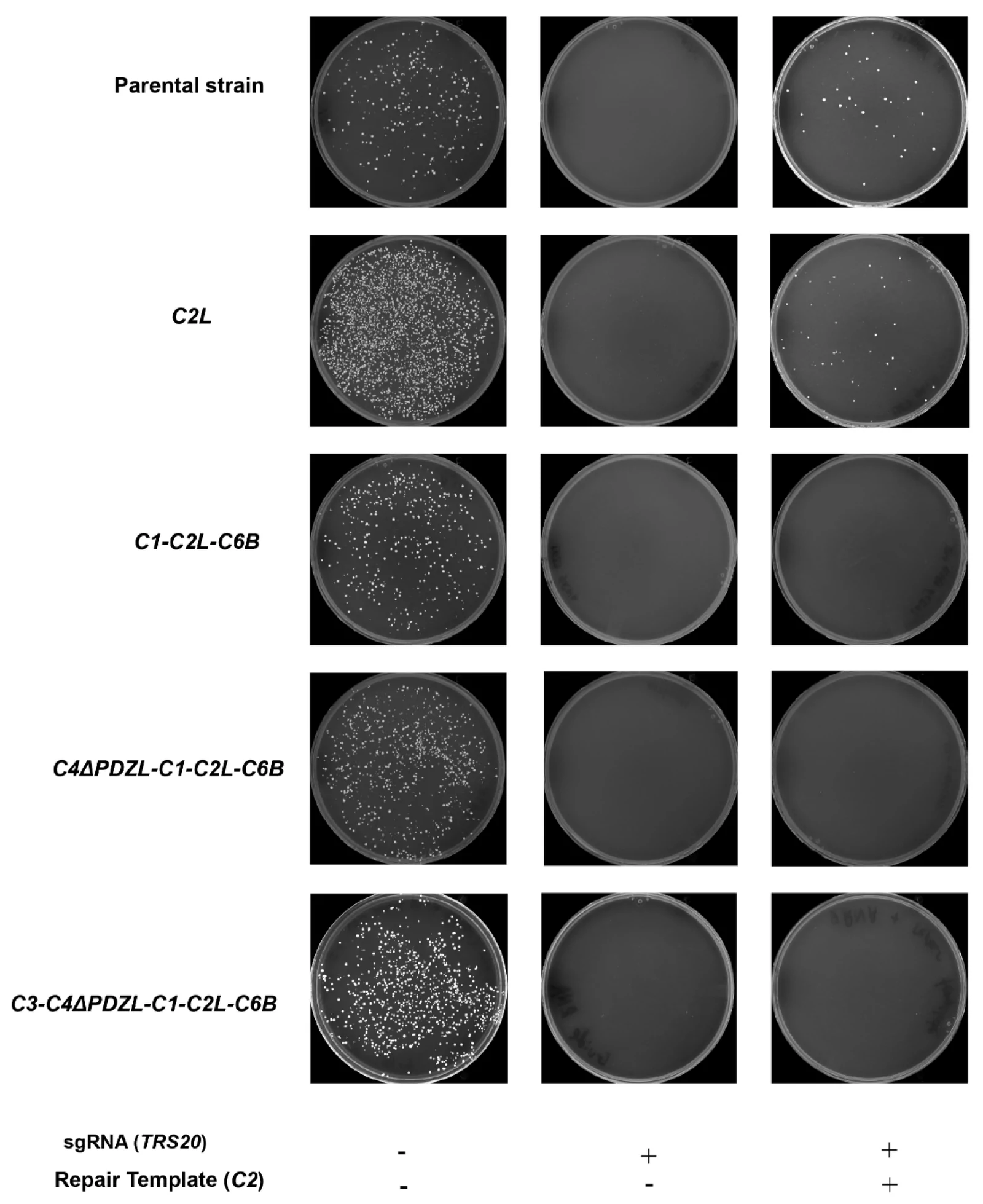
Sequential replacement of *TRS20* with *C2* across intermediate humanized TRAPP strain backgrounds using CRISPR-Cas9. Each row represents a yeast strain expressing an increasing number of human TRAPP subunits, as indicated. CRISPR-Cas9 editing targeted the yeast *TRS20* gene (*C2* ortholog), and the human *C2* ORF was used as the repair template. The presence or absence of sgRNA and repair template is indicated below each column. Transformants were selected on YPD + G418. Colony growth in the +sgRNA/+repair template condition indicates successful replacement of *TRS20* with *C2*. The PHTC result shown in the final row is also presented in Figure S3 as part of the initial analysis of *C2* replacement in the PHTC background and is reproduced here to allow comparison across the sequentially humanized strain series.

Sequential replacement of *TRS20* with *C2* reveals context-dependent compatibility. To better understand the conditions under which *C2* can replace *TRS20*, we performed a sequential replacement approach by introducing C2 into various combinations of humanized strains (Figure 6). The results showed that *C2* could successfully replace *TRS20* only in the parental strain and in a strain in which Tca17 had also been replaced by C2L. In all other humanized combinations tested, C2 replacement was unsuccessful. This demonstrates that the ability of C2 to complement its yeast orthologue is highly context-dependent; it functions in the presence of yeast containing C2L but fails when additional yeast subunits are replaced by their human orthologues. These findings highlight the complexity of multisubunit assembly and suggest that successful integration of certain human subunits may be restricted by the broader composition of the complex.

### 3.3. Structural Modeling and Clash Analysis of PHTC

**Figure 7:**
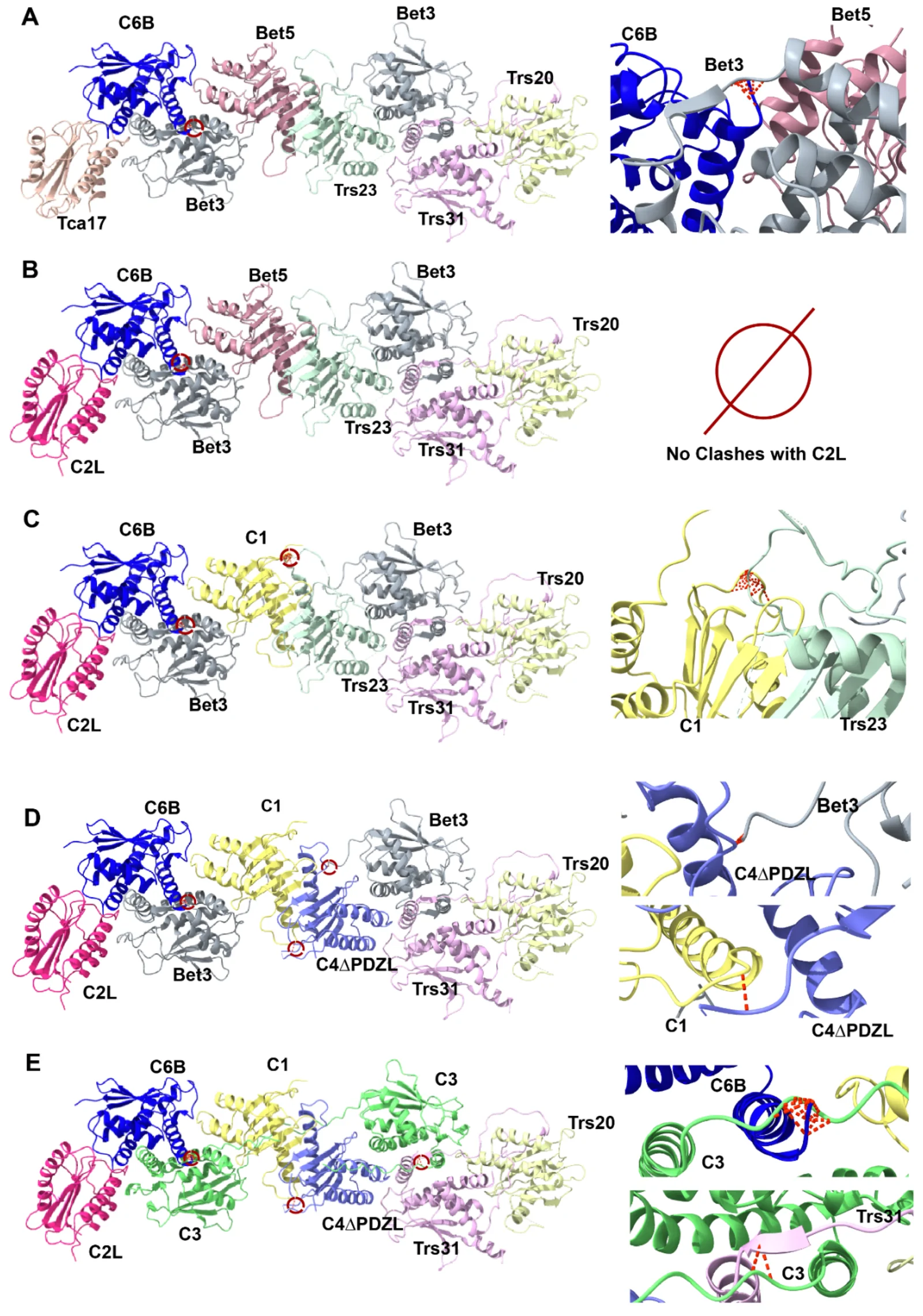
Structural modeling of potential steric clashes during sequential humanization of the TRAPP core. For each panel, the left side shows the overall structure of the TRAPP core complex with the indicated subunit replaced by its human ortholog, and the right side shows a zoomed-in view of the corresponding steric clash. Clash analysis was performed in ChimeraX using the parameters shown (bottom), with only new clashes emerging after the addition of each human subunit displayed. Red circles in the overview panels indicate the position of the clash; zoomed-in views highlight the detailed atomic overlap (red dashed lines). Only clashes appearing after humanization at each stage are shown. (**A**) C6B replaces Trs33. (**B**) C2L replaces Tca17p. (**C**) C1 replaces Bet5. (**D**) C4ΔPDZL replaces Trs23. (**E**) C3 replaces Bet3. Clash detection parameters: VDW overlap ≥ 0.60 Å after subtracting 0.40 Å for H-bonding, ignoring interactions between atoms 4 or fewer bonds apart, and excluding interactions between residues <5 apart in sequence. Both intermodel and intramodel clashes are included. Clashes are displayed as red dashed pseudobonds (radius 0.15, 4 dashes).

To assess how sequential humanization affects structural compatibility among TRAPP subunits, stepwise structural modeling of the humanized strain was performed, reflecting the order of experimental subunit replacement. In the parental strain, no steric clashes were observed (not shown). Replacement of Trs33 with C6B introduced clashes between C6B and Bet5 (Figure 7A). Substitution of Tca17 with C2L did not result in additional clashes (Figure 7B). Subsequent replacement of Bet5 with C1 led to new clashes between C1 and Trs23 (Figure 7C). Introduction of C4ΔPDZL in place of Trs23 generated further clashes, specifically between C4ΔPDZL and C1, as well as C4ΔPDZL and Bet3 (Figure 7D). Finally, replacing Bet3 with C3 resulted in clashes between C3 and C6B and between C3 and Trs31 (Figure 7E). Notably, the clashes previously observed between Bet3 and C4ΔPDZL were no longer present after the replacement with C3. These results demonstrate that progressive humanization of the TRAPP complex can lead to the emergence or resolution of structural incompatibilities, suggesting that the stability of the humanized complex is context-dependent and influenced by the specific combination of subunits present.

### 3.4. Functional Assessment of Humanized Strains by Growth Analysis

To comprehensively assess the general degree of complementation and function of the humanized TRAPP complexes, we measured the fitness of the relevant strains with growth rate analyses. This approach allowed for a robust comparison of the growth characteristics of partially humanized strains relative to the parental yeast strain and to one another.

**Figure 8:**
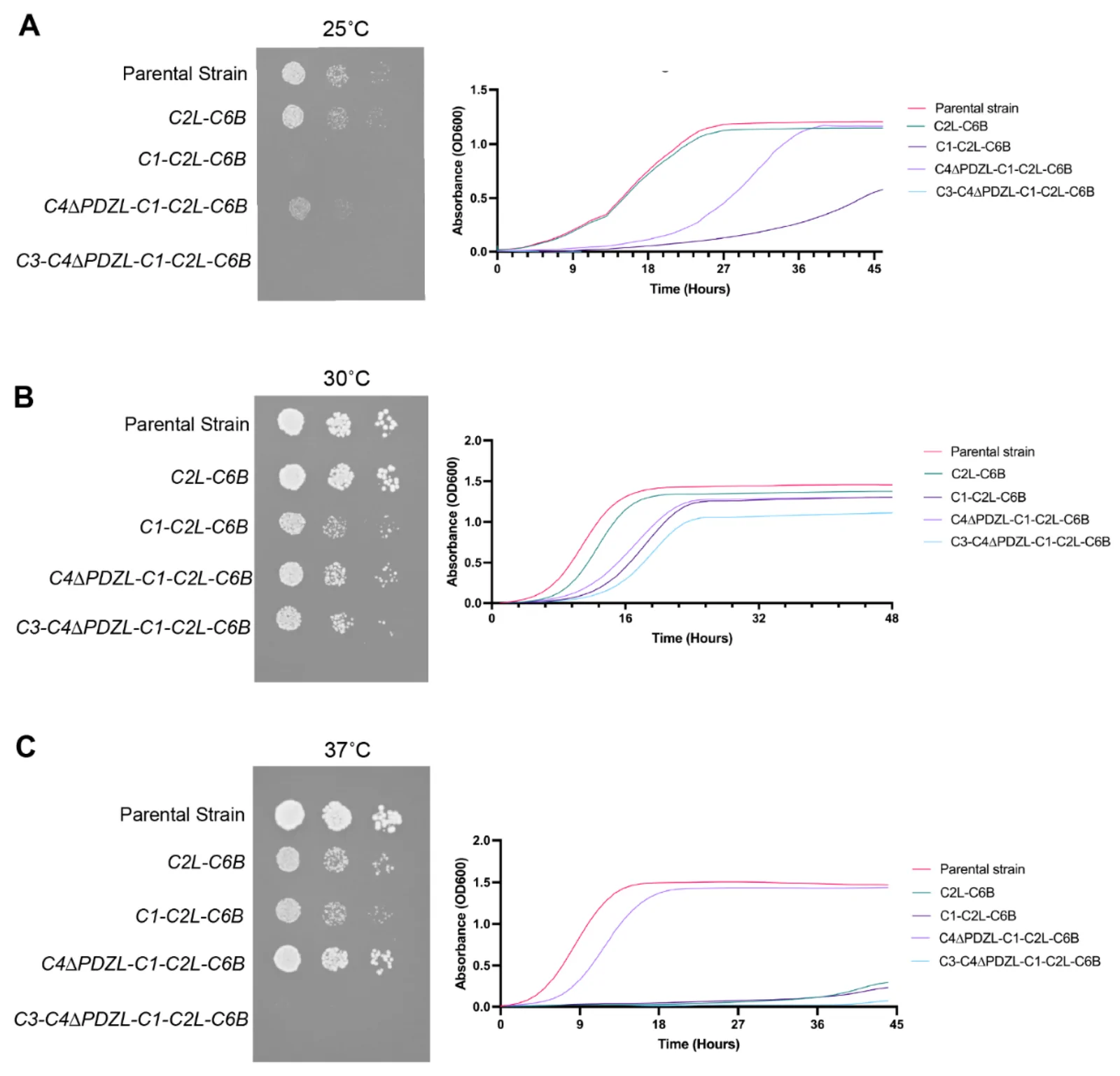
Temperature-dependent growth analysis of sequentially humanized TRAPP core strains. Yeast growth at 25 °C (**A**), 30 °C (**B**), and 37 °C (**C**). Each panel includes a YPD plate spot assay (**left**) and the corresponding liquid growth curve (**right**). For the spot assays, three 10-fold serial dilutions of each indicated strain were plated on YPD and incubated at the indicated temperature. Data are presented as the mean of three biological replicates, each with three technical replicates, all showing the same growth pattern. In parallel, liquid cultures of the same strains were grown under identical conditions, and growth was monitored by measuring OD_600_ over time.

At both 25 °C and 30 °C, the strain harboring humanized *C2L* and *C6B* exhibited growth indistinguishable from that of the parental strain, suggesting that these two human subunits are well tolerated and integrate compatibly within the largely yeast TRAPP complex (Figure 8A). However, when challenged at 37 °C, this strain displayed a clear reduction in growth and increased temperature sensitivity, indicating reduced functional compatibility under temperature stress. The addition of C1 (C1-C2L-C6B) resulted in a strain that grew more poorly than either the parental or the double humanized C2L-C6B strain across all three tested temperatures, indicating that introduction of C1 reduces functional complementation in this humanized background.

Interestingly, the strain containing C4ΔPDZL-C1-C2L-C6B reached a delayed growth plateau at 25 °C and 30 °C, but unexpectedly displayed growth comparable to the parental strain at 37 °C. This finding suggests that removal of the PDZL domain from C4 may enhance its compatibility with the yeast complex, resulting in improved functional compatibility at higher temperatures where other humanized strains falter. In stark contrast, the PHTC strain containing C3-C4ΔPDZL-C1-C2L-C6B failed to grow at both 25 °C and 37 °C, and exhibited the most impaired growth at 30 °C. This severe fitness defect highlights the cumulative challenge of assembling multiple human subunits within the yeast TRAPP complex and underscores the limitations imposed by structural incompatibility.

### 3.5. Successful Replacement of BET3 with the C3 p.(R62W), p.(L131F), and p.(R164W) Variants

**Figure 9:**
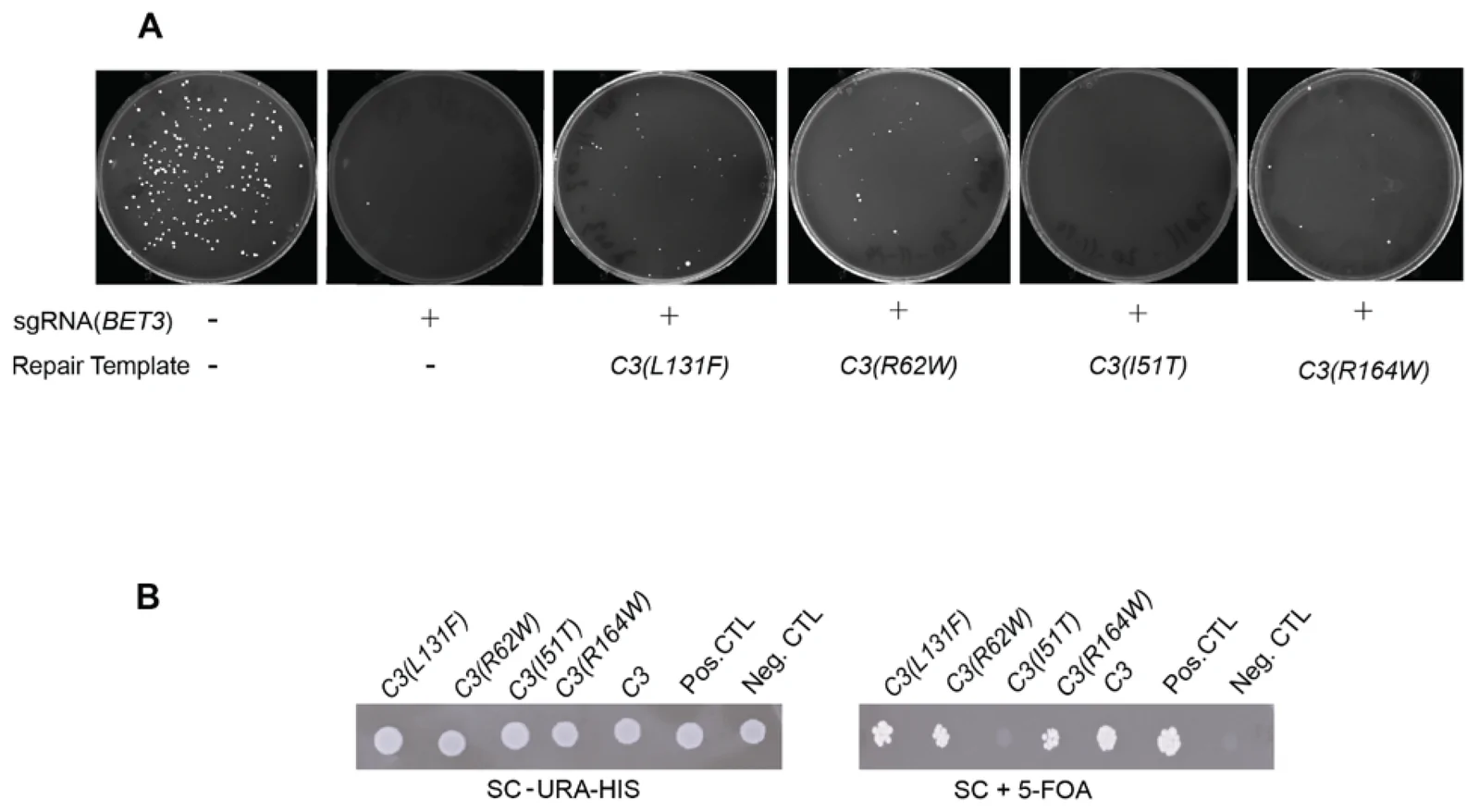
Successful replacement of *BET3* with three *C3* variants in the PHTC strain. (**A**) Yeast transformation plates showing the CRISPR-Cas9-mediated replacement of *BET3* with four human *C3* variants indicated. The empty-plasmid and sgRNA-only control plates are the same experimental controls shown in Figure 3B and are reproduced here for completeness as part of the larger analysis of *C3* variants. Colonies on YPD+G418 selection plates are observed on all plates except for the I51T variant, indicating successful integration of the human C3 variants. (**B**) A *bet3*Δ yeast strain harboring a *URA3*-based plasmid expressing *BET3* was transformed with *HIS3*-based plasmids expressing wild-type C3 for each of the four C3 variants indicated. The strains were then grown on either SC-Ura-Leu or 5-FOA plates. Positive (*BET3* on a *HIS3*-based plasmid) and negative (empty plasmid) controls were also included.

One of the goals of this work was to establish a simple and inexpensive yeast platform for functional analysis of human TRAPP variants of uncertain significance (VUS), particularly when patient-derived cells are unavailable. To this end, we examined four homozygous VUS in C3, which has not yet been definitively shown to be associated with human disease. The variants were assessed using the PHTC yeast model. They included C3p.(R62W) [29], identified as a putative cause of disease in two siblings from Saudi Arabia investigated for a Bardet Biedl syndrome (BBS) phenotype as part of a larger ciliopathy disorder cohort study, p.(I51T); DECIPHER ID: 294488, and p.(L131F); DECIPHER ID: 277579, identified through the Deciphering Developmental Disorders Study in two individuals affected by an undiagnosed developmental disorder and some features compatible with a ciliopathy, and lastly p.C3(R164W), which was identified through clinical testing of a Palestinian individual with growth and developmental impairment. We used the partially humanized strain (*C4∆PDZL*-*C1*-*C2L*-*C6B*) to introduce all C3 mutants. A summary of the VUS is presented in Table S1. As shown in Figure 9A, only the p.(R62W), p.(L131F), and p.C3(R164W) mutants resulted in viable yeast. Sanger sequencing (Figure S4) further confirmed the integration of these variants. This demonstrates that three of the four mutations are tolerated in the context of the humanized TRAPP complex in yeast. Speculating that the inability of the C3p.(I51T) mutant to give viable yeast was due to insufficient expression levels, we set up a plasmid shuffle experiment where a *bet3*Δ strain was kept alive with a balancing *URA3*-based plasmid containing *BET3*. The strain was then transformed with a *LEU2*-based plasmid containing all of the C3 mutants under a strong *ADH1* promoter and plated on FOA-containing plates. These plates counter-select for the *URA3*-based plasmid. While wild-type C3 and all of the viable C3 variants grew on FOA plates, the C3p.(I51T) mutant did not (Figure 9B). This suggests that even when overproduced, this particular variant is inviable in yeast.

**Figure 10:**
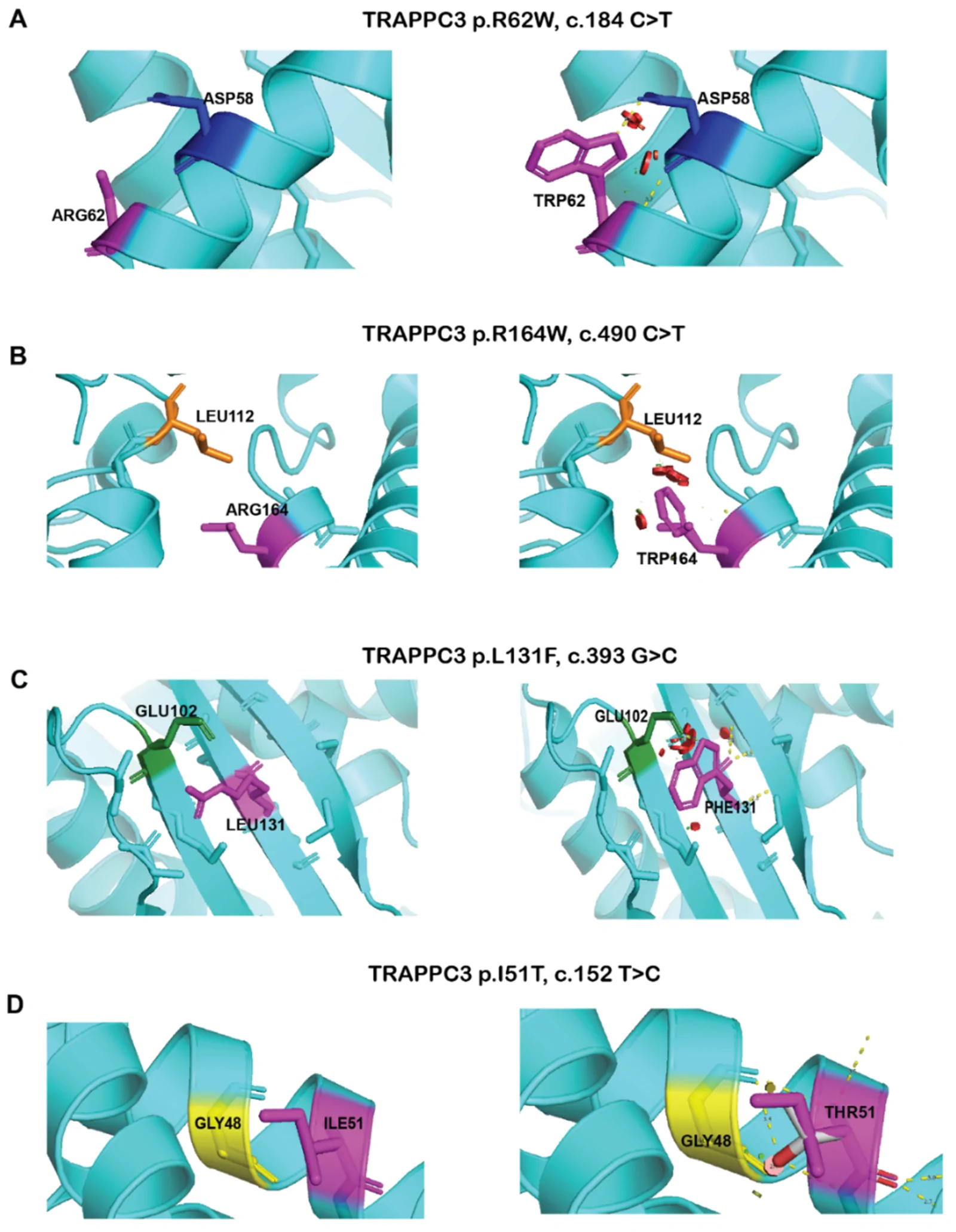
Structural and sequence analysis of the C3 mutants and their effects on protein packing. Each of the four C3 variants, as indicated, was modeled, and clash analysis was performed using PyMOL and verified with Chimera. In each of the panels, the left side indicates the wild-type residue and its local region, while the right side indicates the C3 variant. Clashes are indicated by the red objects and are only seen in the C3 variants.

To predict the structural consequences of these mutations, wild-type (WT) and mutant C3 sequences were modeled and analyzed using PyMOL. Residue interactions were evaluated based on van der Waals clashes, using a 0.3 Å cutoff. This analysis illustrates that in the WT structure, residue Leu131 showed no steric clashes with adjacent residues (Figure 10). However, in the mutant structure, the substitution of Leu131 to Phe131 introduced a clash with residue Glu102. This clash was consistently identified in both PyMOL and Chimera analyses, reinforcing the reliability of this structural prediction. Similarly, substitution of Arg62 with Trp introduced a clash with Asp58, substitution of Arg164 with Trp introduced a clash with Leu112 and substitution of Ile51 with Thr introduced a clash with Gly48. These results suggest that these mutations introduce steric hindrance in the C3 structure, potentially affecting the structural stability or interaction profile within the TRAPP complex.

### 3.6. Characterization of PHTC Strains Containing C3 Variants of Uncertain Significance

**Figure 11:**
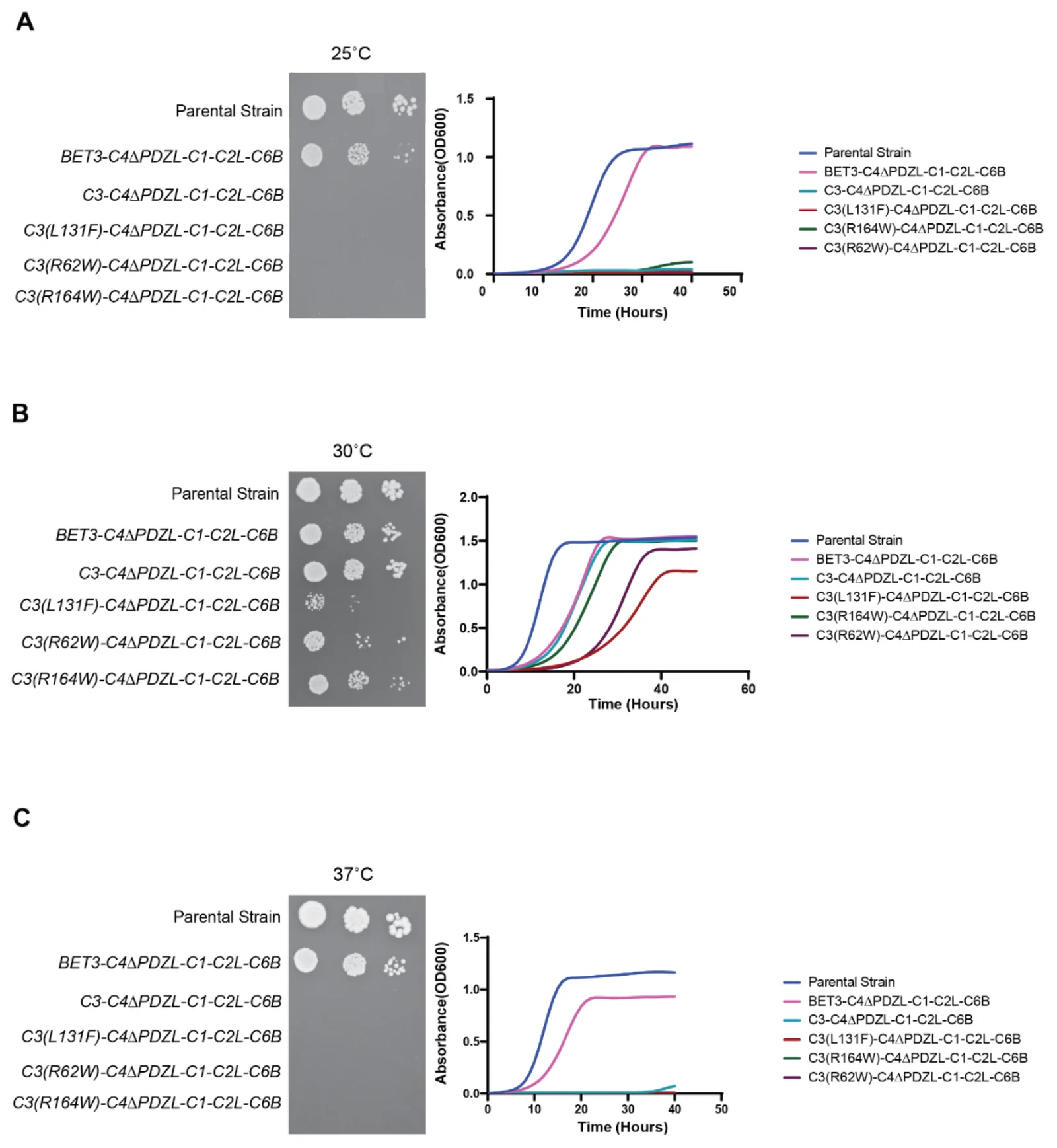
Growth analysis of parental, C3 wild-type, and C3 variant strains at different temperatures. Panels (**A**–**C**) show serial dilution spot assays (**left**) and corresponding growth curves (**right**) at 25 °C, 30 °C, and 37 °C, respectively. Each assay includes the parental strain, a strain containing wild-type C3 in the PHTC background, and a strain containing the C3 variants indicated in the same background. Data are presented as the mean of three biological replicates, each with three technical replicates, all showing the same growth pattern.

To investigate the functional consequences of the three viable C3 variants, we compared the growth of the parental strain, PHTC and strains containing each of the three C3 variants across a range of temperatures. Growth was assessed both by spot assays and by monitoring liquid culture growth curves at 25 °C, 30 °C, and 37 °C (Figure 11). At all three temperatures, the paternal strain showed robust growth. The strain with C1-C2L-C6B-C4ΔPDZL also showed growth at all temperatures but slightly reduced compared to wild type. Introduction of C3 to this strain (i.e., PHTC) abolished growth at low (25 °C) and high (37 °C) temperatures. However, growth of this strain slightly exceeded that of the strain with Bet3, indicating that replacement of *BET3* with *C3* improved growth relative to the C1-C2L-C6B-C4ΔPDZL strain under this condition. Humanization of the *BET3* locus with the three functional C3 variants reduced the growth of the respective strains compared to PHTC at 30 °C and, like wild-type C3, abolished growth at lower and higher temperatures. The pronounced growth defects of the C3 variants indicate that these variants compromise the ability of human C3 to functionally substitute for yeast Bet3 in the PHTC background.

## 4. Discussion

This study expands the utility of the humanized yeast model as a platform for investigating multisubunit complexes by enabling the incorporation of human TRAPP subunits that could not previously substitute individually for their yeast orthologues. Using scarless CRISPR-Cas9 editing while preserving endogenous yeast regulatory elements, we expressed several human TRAPP subunits from the corresponding endogenous yeast loci. Building on prior work that validated this model for functional analysis of VUS [24], we extended its scope to address more challenging targets, such as C3 and a truncated form of C4. The ability to incorporate these human subunits into the yeast TRAPP core highlights the flexibility of this model and reinforces its potential for rapid, in vivo assessment of TRAPP-related VUS. However, certain subunits, such as C5, remained refractory to replacement, underscoring the complex interplay of structural compatibility within conserved assemblies.

The successful stepwise replacement of TRAPP subunits such as C6B, C2L, and C1 demonstrates that the yeast system can accommodate multiple human proteins simultaneously, supporting its utility as a platform for progressively assembling multisubunit human complexes. This is also consistent with large-scale efforts to humanize conserved genes individually [5]. However, not all subunits proved readily replaceable. The inability of human *C4* to replace yeast *TRS23* under standard conditions underscores a broader challenge in humanizing multisubunit complexes, namely the need for precise structural compatibility at subunit interfaces. This issue has been observed in other systems as well. For example, it was shown that successful humanization of the proteasome β2 subunit required appending a yeast-specific C-terminal tail to maintain inter-subunit contacts [10]. Analogously, our comparative analysis revealed that human C4 contains a PDZL domain absent in yeast Trs23, potentially disrupting interactions within the TRAPP core. By removing this domain, we generated a truncated variant (C4ΔPDZL) that restored functionality in yeast, suggesting that domain-specific incompatibilities, rather than broader sequence divergence, can be key determinants of cross-species replacement success. C4ΔPDZL was generated specifically as an engineered yeast-compatible derivative, and its ability to functionally substitute for Trs23 should not be interpreted as demonstrating physiological equivalence to full-length TRAPPC4 in mammalian cells. These findings highlight the importance of structural tailoring in overcoming evolutionary barriers during humanization efforts.

The inability of C3 to substitute for Bet3 in isolation, despite its high sequence similarity to yeast Bet3 [24], suggests that sequence identity alone is insufficient for functional replacement within multisubunit assemblies. This result highlights the importance of structural context and inter-subunit dependencies during humanization. C3 occupies two distinct positions within the TRAPP core, one of which interacts with C4 and C1 while the other interacts with C5 and C2, implying that its successful incorporation may require compatible interfaces with neighboring proteins. Replacement of *BET3* with *C3* was successful when both C1 and the truncated C4ΔPDZL were present, consistent with the possibility that increased compatibility with neighboring subunits facilitates its functional integration. These findings support the idea that successful replacement in conserved complexes often depends on cooperative interactions among subunits, rather than the properties of an individual subunit in isolation. This was also observed in humanization efforts where context-dependent compatibility was shown to influence success [3].

Growth phenotypes in humanized yeast do not correlate directly with the number of human subunits present, but rather appear to reflect the specific functional compatibility of the human subunits within the yeast TRAPP complex. For example, strains containing both C2L and C6B maintain wild-type growth at 25 °C and 30 °C, indicating effective functional complementation under standard conditions. However, reduced fitness at 37 °C indicates that this functional compatibility is diminished under thermal stress. In contrast, the C4ΔPDZL-containing strain exhibits robust growth across all temperatures, with particularly enhanced growth at 37 °C, suggesting that removal of the PDZL domain improves the ability of human C4 to function in the partially humanized yeast background. Structural modeling provides a possible explanation for this observation, as inclusion of the PDZL domain introduces additional steric clashes with C3. Conversely, replacement of *BET3* with *C3* leads to consistently lower growth at all tested temperatures, indicating reduced functional complementation in this background. More broadly, structural analysis reveals that introducing additional human subunits can lead to the emergence or resolution of steric clashes, depending on the particular combination of subunits present. Together, these findings suggest that growth phenotypes in humanized yeast are strongly influenced by the specific compatibility of interacting human and yeast TRAPP subunits rather than simply by the extent of humanization.

These incompatibilities may explain why *C2* can successfully replace *TRS20* when introduced into an otherwise yeast TRAPP background, yet fails to do so when additional human subunits are present. This observation suggests that successful replacement depends not only on sequence homology but also on the particular subunit context in which the human protein is introduced. In the case of C2, its ability to integrate functionally depends not only on its own properties but also on the specific combination and context of its interaction partners. These findings emphasize that humanization of multisubunit assemblies requires consideration of both sequence similarity and structural context, particularly as the number of humanized subunits increases.

This study demonstrates the utility of the humanized yeast platform for functional interrogation of TRAPP variants and provides evidence that the four C3 VUS impair the ability of human C3 to functionally substitute for yeast Bet3. Growth represents an integrated cellular phenotype and does not identify the specific molecular process responsible for reduced fitness. Thus, although reduced growth provides a measure of impaired functional complementation by a human TRAPP protein or variant, the present experiments do not establish whether the phenotype results specifically from altered vesicular trafficking, Rab activation, TRAPP complex assembly, or another downstream consequence of impaired TRAPP function. Direct analysis of these processes will be important in future mechanistic studies. Although the human ORFs were introduced at the endogenous yeast loci, steady-state abundance of the human proteins was not quantitatively compared with that of their yeast orthologues. Differences in translation efficiency or protein stability could therefore contribute to some of the observed growth phenotypes. This approach supports the use of humanized yeast models for assessing VUS and generating functional evidence to inform variant interpretation.

A further limitation is that the humanized strains were not subjected to genetic segregation analysis or whole-genome sequencing to formally exclude spontaneous second-site mutations acquired during strain construction. Although the rapid recovery of transformants and the reproducible context- and allele-dependent phenotypes observed across the humanization series argue against adaptive mutations as the primary explanation for our findings, future studies using independently derived isolates, segregation analysis, or whole-genome sequencing could address this possibility directly.

It was noteworthy that one of the *C3* VUS (p.I51T; c.152 T>C) was non-functional in yeast while the others displayed reduced functionality, yet all four introduce localized amino acid clashes. The I51T variant affects a residue lining the C3 hydrophobic tunnel that accommodates palmitic acid [30,31]. The structurally related C6B protein lacks this tunnel and instead has bulky hydrophobic residues in the region where the tunnel would be [32]. This has led to the speculation that the palmitic acid contributes to the overall structural stability of C3 [33]. Replacement of the hydrophobic isoleucine with a polar threonine in the center of the tunnel would be predicted to constrict the middle of the tunnel, thus precluding full insertion of the hydrophobic lipid. This is expected to have profound effects on the structure and/or stability of C3, possibly explaining the inability of this variant to function in yeast. Consistent with this notion, we have found that recombinant C3p.I51T is poorly expressed compared to wild type or either of the other three variants (unpublished observation). Thus, clash analysis alone may not fully predict function and protein stability in humanization models.

*TRAPPC3* was proposed as a potential new TRAPPopathy disease gene by Shaheen et al. following identification of the homozygous NM_014408.5(TRAPPC3):c.184C>T (p.Arg62Trp) variant in two siblings with classical BBS clinical features [29]. Since publication of the original findings, copy number variation (CNV) analysis has identified a possible duplication in *TTC8*. Biallelic pathogenic *TTC8* variants are an established cause of BBS. It has not been possible to confirm the *TTC8* duplication; as such, the potential clinical relevance of this and the pathogenicity of the *TRAPPC3* variant identified in this family remain unclear. No further candidate *TRAPPC3* variants have been published since this time.

Although functional defects observed in the humanized yeast model provide strong evidence that a variant disrupts TRAPP function, they do not, on their own, establish disease–gene association or causality. Rather, these findings represent one line of functional evidence that should be integrated with clinical, genetic, and additional experimental data, including segregation analyses, population frequency, and validation in mammalian or patient-derived systems, to support identification of new TRAPPopathy disorders and classification of variant pathogenicity. Further genomic and clinical studies are required to determine a TRAPPC3 gene–disease association. However, within this framework, the humanized yeast platform provides an ideal system for high-throughput functional assessment of variants generated by site-directed mutagenesis, enabling systematic evaluation of their effects on complex assembly, protein stability, and cellular growth. Future studies combining this platform with biophysical characterization of purified TRAPP complexes and collaborative clinical genetic analyses will further strengthen genotype–phenotype interpretation.

## 5. Conclusions

This study demonstrates that stepwise humanization of the yeast TRAPP core can overcome incompatibilities that prevent individual human subunits from functionally replacing their yeast orthologues. The ability of a human subunit to function in yeast depends strongly on the composition of the surrounding complex, highlighting the importance of protein–protein interactions and structural context when humanizing multisubunit assemblies. The resulting partially humanized TRAPP core provides a tractable system for assessing the functional consequences of human TRAPP variants, as demonstrated using C3 variants of uncertain significance. While such functional data cannot alone establish variant pathogenicity or disease–gene association, this platform provides a rapid and scalable approach for generating experimental evidence to support variant interpretation and the study of TRAPPopathies.

## Figures and Tables

**Table 1:** Plasmids used in this study.

Plasmid	Backbone	Source/Accession Number
MSB26	pRS426 *TRS33*	Sacher laboratory
MSB 33	pRS316 *BET3*	Sacher laboratory
MSB 41	pRS426 *TRS23*	Sacher laboratory
MSB 400	pRS413-ADH1pr	Sacher laboratory
MSB 579	pDONR201 *TRAPPC1*	Sacher laboratory/NM_021210.5
MSB 580	pDONR201 *TRAPPC3*	Sacher laboratory/NM_014408.5
MSB 581	pDONR201 *TRAPPC4*	Sacher laboratory/NM_016146.6
MSB 582	pDONR201 *TRAPPC2L*	Sacher laboratory/NM_016209.5
MSB 586	pDONR201 *TRAPPC2*	Sacher laboratory/NM_014563.5
MSB 607	pGADT7-GWY *TRAPPC6B*	Sacher laboratory
MSB 612	pDONR201 *TRAPPC5*	Sacher laboratory/NM_174894.3
MSB 1258	pRS426 *BET3*	Sacher laboratory
MSB 1805	pCEN6-Cas9-GFP-KanMX	Kachroo laboratory
MSB 1806	pCEN6-Cas9-GFP-KanMX *BET5* sgRNA1	Zykaj et al. [24]
MSB 1820	pCEN6-Cas9-GFP-KanMX *TRS20* sgRNA1	Zykaj et al. [24]
MSB 1821	pCEN6-Cas9-GFP-KanMX *TRS23* sgRNA1	Zykaj et al. [24]
MSB 1823	pCEN6-Cas9-GFP-KanMX *BET3* sgRNA1	Zykaj et al. [24]
MSB 1824	pCEN6-Cas9-GFP-KanMX *TRS31* sgRNA1	Zykaj et al. [24]
MSB 1826	pCEN6-Cas9-GFP-KanMX *TCA17* sgRNA1	Zykaj et al. [24]
MSB 2034	pRS413-ADH1pr-TRAPPC3 (L131F)	This study
MSB2037	pRS413-ADH1pr-TRAPPC3 (R62W)	This study
MSB 2039	pRS413-ADH1pr-TRAPPC3 (R164W)	This study
MSB 2042	pRS413-ADH1pr-TRAPPC3	This study
MSB 2044	pRS413-ADH1pr-TRAPPC3 (I51T)	This study
MSB 2046	pCEN6-Cas9-GFP-KanMX *C4∆PDZL* sgRNA2	This study

**Table 2:** Yeast strains used in this study.

Yeast Strain	Genotype	Source
MSY 76	*MATα his3∆1 leu2∆0 ura3∆0* with *pRS316-BET3*	Sacher laboratory
BY4742	*MATα his3∆1 leu2∆0 lys2∆0 ura3∆0*	Brachmann et al. [25]
MSY 988	*MATα his3∆1 leu2∆0 lys2∆0 ura3∆0 trs33∆::C6B*	This study
MSY 1014	*MATα his3∆1 leu2∆0 lys2∆0 ura3∆0 tca17∆::C2L trs33∆::C6B*	This study
MSY 1027	*MATα his3Δ1 leu2Δ0 lys2Δ0 ura3Δ0 tca17∆::C2L trs33∆::C6B bet5∆::C1*	This study
MSY 1047	*MATα his3Δ1 leu2Δ0 lys2Δ0 ura3Δ0 tca17∆::C2L trs33∆::C6B bet5∆::C1 trs23∆:: C4∆PDZL*	This study
MSY 1060	*MATα his3Δ1 leu2Δ0 lys2Δ0 ura3Δ0 tca17∆::C2L trs33∆::C6B bet5∆::C1 trs23∆:: C4∆PDZL bet3∆::C3(L131F)*	This study
MSY 1061	*MATα his3Δ1 leu2Δ0 lys2Δ0 ura3Δ0 tca17∆::C2L trs33∆::C6B bet5∆::C1 trs23∆:: C4∆PDZL bet3∆::C3*	This study
MSY 1063	*MATα his3Δ1 leu2Δ0 lys2Δ0 ura3Δ0 tca17∆::C2L trs33∆::C6B bet5∆::C1 trs23∆:: C4∆PDZL bet3∆::C3(R164W)*	This study
MSY 1069	*MATα his3Δ1 leu2Δ0 lys2Δ0 ura3Δ0 tca17∆::C2L trs33∆::C6B bet5∆::C1 trs23∆:: C4∆PDZL bet3∆::C3(R62W)*	This study
MSY 1098	*MATα his3Δ1 leu2Δ0 lys2Δ0 ura3Δ0 pRS426-BET3*	Sacher laboratory
MSY 1099	*MATα his3Δ1 leu2Δ0 lys2Δ0 ura3Δ0 pRS426-TRS33*	Sacher laboratory
MSY 1100	*MATα his3Δ1 leu2Δ0 lys2Δ0 ura3Δ0 pRS426-TRS23*	Sacher laboratory

**Table 3:** Confirmation PCR primers.

Primer Name	Sequence
*TRAPPC1*-Fp	GTCCTTTACTATTGACTAGTG
*TRAPPC1*-Rp	CATCCCGTACATCAGCTTATAC
*TRAPPC3*-Fp	ACTCCAAGCCAAAGGTAAGG
*TRAPPC3*-Rp	GCCAAGAAATCTTCAATCAGC
*TRAPPC2L*-Fp	AAGTTCTGCCGGAAGAGCTCATC
*TRAPPC2L*-Rp	ATTGCGGAGATCTTCTCATCC
*TRAPPC6B*-Fp	GGAATCTGGTATGCACTCAC
*TRAPPC6B*-Rp	AACTCATCCTTGAACCTTGCAG
*TRAPPC4*-Fp	TGTTGGAGTTTATGCCTCCTC
*TRAPPC4*-Rp	GGTCCAAAAGTTCCAGCCTT

**Table 4:** List of primers used for sequencing.

Sequencing Oligos	Sequence
BET5seq-F	CTGGTTTGCCTCAGTCGAAATG
BET5seq-R	CCCAAACCAGATCCGAGTTTG
TRS20seq-F	CCTCCTCTGCTGAAGTGTAT
TRS20seq-R	GTAACGGAACACTCAAAAGTG
TCA17seq-F	TCACTTTCGAAGTTCTGCCG
TCA17seq-R	ATATACTACCGGCACCTGATC
TRS33seq-F	CATTAGTAGAACCAGCGTATCG
TRS33seq-R	GGCTTTAAGAAGAGTCCACGA
BET3seq-F	CGAAACTCCAAGCCAAAGGTAAG
BET3seq-R	ATTGCTTCGCTGGTAAGACAG

**Table 5:** List of sgRNAs targeting the genes in yeast.

sgRNA Oligos	Sequence
*BET5* sgRNA1-Fp	GACTTTTTACACAAAGGCTCTCCAAA
*BET5* sgRNA1-Rp	AAACTTTGGAGAGCTTTTGTGTAAAA
*BET5* sgRNA2-Fp	GACTTTGGCTGTTTAAAATGACATA
*BET5* sgRNA2-Rp	AAACATGTCAATTTTAAACAGCCAA
*TRS20* sgRNA1-Fp	GACTTTACCAATGCAGAAATCCACA
*TRS20* sgRNA1-Rp	AAACGTGGATTTTCTGCATTGGTA
*TRS20* sgRNA2-Fp	GACTTTAGTGATTGCATGCAGTAGAA
*TRS20* sgRNA2-Rp	AAACATTCATACGCATGCATCACTA
*TCA17* sgRNA1-Fp	GACTTTGATTTACTGCCCCTAATGAGG
*TCA17* sgRNA1-Rp	AAACCCTCATTAGGGGCAAGTTAACAA
*TCA17* sgRNA2-Fp	GACTTTTCTGAAGTCAATTTTCAAT
*TCA17* sgRNA2-Rp	AAACATTGAAAAATTGACTTCAGAAA
*BET3* sgRNA1-Fp	GACTTTCTTCAAGACCTGCATTGCG
*BET3* sgRNA1-Rp	AAACCGCAATCGAGGCTCTTTGAAGAA
*BET3* sgRNA2-Fp	GACTTTGCTTCAATATGGGCTGCATA
*BET3* sgRNA2-Rp	AAACCATATGCAGCCCATATTGAAGC
*TRS23* sgRNA1-Fp	GACTTTCTTGTAATAAACAAATCAGG
*TRS23* sgRNA1-Rp	AAACCCGTATTGTTTTATTACAAGAA
*TRS23* sgRNA2-Fp	GACTTTATTCTTGCTAGTACACTGCA
*TRS23* sgRNA2-Rp	AAACGTGCAGTACTAGCAAGAATAAA
*TRS31* sgRNA1-Fp	GACTTTGCACTGCACCAACAATTCCC
*TRS31* sgRNA1-Rp	AAACGGGAATTGTTGGTGCAGTGCAA
*TRS31* sgRNA2-Fp	GACTTTAGAAGCGCTCTTTATCAGCAA
*TRS31* sgRNA2-Rp	AAACCTGCGATAAAGAGCGCTTCTAA
*TRS33* sgRNA1-Fp	GACTTTGCAGCCTGTGGGAAAGATTG
*TRS33* sgRNA1-Rp	AAACCAATTCTTCCACAAAGGTGCAG
*TRS33* sgRNA2-Fp	GACTTTCTCAGCTAGTGGGCATGGAG
*TRS33* sgRNA2-Rp	AAACCTCAATGGCCCATAGCTAGAGA
*C4-pdzl* sgRNA1-Fp	TTACCAGTTGGACAGCTACG
*C4-pdzl* sgRNA1-Rp	CGTAGCTGTCCAACTGGTAA
*C4-pdzl* sgRNA2-Fp	TTCTAATGAGAAGCTTATGC
*C4-pdzl* sgRNA2-Rp	GCATAAGCTTCTCATTAGAA

**Table 6:** Oligonucleotides used for TRAPPC3 mutagenesis.

Primer Name	Sequence
TRAPPC3 I51T UP	CAAAATGGGCTTTAACACTGGAGTCCGGCTG
TRAPPC3 I51T DN	CAGCCGGACTCCAGTGTTAAAGCCCATTTTG
TRAPPC3 R62W UP	GAAGATTTCTTGGCTTGGTCAAATGTTGGG
TRAPPC3 R62W DN	CCCAACATTTGACCAAGCCAAGAAATCTTC
TRAPPC3 L131F UP	CTTATTTATTCCAATCTCTTCTGTGGGGTGTTGCGG
TRAPPC3 L131F DN	CCGCAACACCCCACAGAAGAGATTGGAATAAATAAG
TRAPPC3 R164W UP	GGTGTGACAGAAATCTGGATGAGATTCATC
TRAPPC3 R164W DN	GATGAATCTCATCCAGATTTCTGTCACACC

**Table 7:** Quantitative RT-PCR primers.

qRT-PCR Primer	Sequence
*C1* F	CTCTATCCGCTCGTTTGTCAGC
*C1* R	CCACGCCCAAGTCAGTATTCATG
*C2L* F	GGTGGTAGATTCCTCCAACACAG
*C2L* R	CGTCACCATGTTATCAAAGGCCC
*C4* F	ACGATGAGCGTGTGTTGGTTGC
*C4* R	CAGATACTCCAGCACCTCTTTCC
*C6B* F	GCCTGCTTACTCAGATGTCTGC
*C6B* R	CAAGCAGGCATTGAAGACACTTC

## Data Availability

The raw data supporting the conclusions of this article will be made available by the authors on request.

## Supplementary Materials

The following supporting information can be downloaded at: https://www.mdpi.com/article/10.3390/cells15171555/s1, Table S1. *TRAPPC3* variants of uncertain significance included in this study. Abbreviations—Hom, homozygous; MAF, minor-allele frequency. Figure S1. Sanger sequencing of C1, C2L, and C6B. Genomic DNA from each humanized yeast strain was PCR-amplified and subjected to Sanger sequencing. The red boxes indicate the start and stop codons of the integrated human open reading frames (ORFs), while the underlined regions correspond to the upstream and downstream untranslated regions (UTRs) of the endogenous yeast genes. These chromatograms confirm scarless replacement of the yeast ORFs with their human counterparts, validating precise genomic integration for each construct. Figure S2. Verification of successful C4∆PDZL and C3 scarless replacement via Sanger sequencing. Chromatograms from Sanger sequencing confirm the seamless integration of human C4∆PDZL and C3 ORFs into the yeast genome. The sequences show the expected junctions between yeast 5′ and 3′ untranslated regions (UTRs) and the inserted human ORFs, demonstrating scarless replacement. For C4∆PDZL, the characteristic ∆PDZL region is indicated. Figure S3. CRISPR-Cas9-mediated humanization of C5 and C2 in a PHTC background. All transformations were performed in the PHTC strain. The top panel shows targeting of the TRS31 gene using a sgRNA and a repair template encoding C5. The bottom panel shows targeting of the TRS20 gene using a sgRNA and a repair template encoding C2. No colonies are observed in the plate that contains the sgRNA with the repair template, indicating unsuccessful replacement. Figure S4. Sanger sequencing confirmation of *C3* variants in the humanized yeast strain The chromatograms show successful integration of the indicated *C3* variant ORFs. The boxed base indicates the nucleotide change introduced by CRISPR-Cas9-mediated genome editing.
