# Supplementary material for: Stepwise Humanization of the Yeast TRAPP Core Enables Functional Analysis of TRAPP Variants
Source: Cells. 2026 Aug 27;15(17):1555. doi: 10.3390/cells15171555 (PMC13564602; doi:10.3390/cells15171555)
Supplement: Supplementary file 1 [file cells-15-01555-s001.zip › cells-4511050-supplementary.pdf]

Table S1. *TRAPPC3* variants of uncertain significance included in this study. Abbreviations - Hom, homozygous; MAF, minor-allele frequency.

Genomic location: (GRCh38)	c. NM_014408.5 (MANE select)	p. NP_055223.1	Zygosity	GnomAD v2.1.1 MAF	GnomAD v4.1.1 MAF	No. of homs gnomAD v4.1.1	*In silico* predictions		
							Revel	Alpha Missense	SpliceAI
Chr1:g.36139776G>A	c.184C>T	p.(Arg62Trp)	Hom	0.00001768	0.00001673	0	0.519	0.902	0.17
Chr1:g.36139808A>G	c.152T>C	p.(Ile51Thr)	Hom	0	0.000002478	0	0.797	0.814	0.02
Chr1:g.36137826C>A	c.393G>T	p.(Leu131Phe)	Hom	0.0001697	0.0003190	0	0.294	0.328	0.03
Chr1:g.36137256G>A	c.490C>T	p.(Arg164Trp)	Hom	0.000003978	0.000003719	0	0.489	0.816	0.00

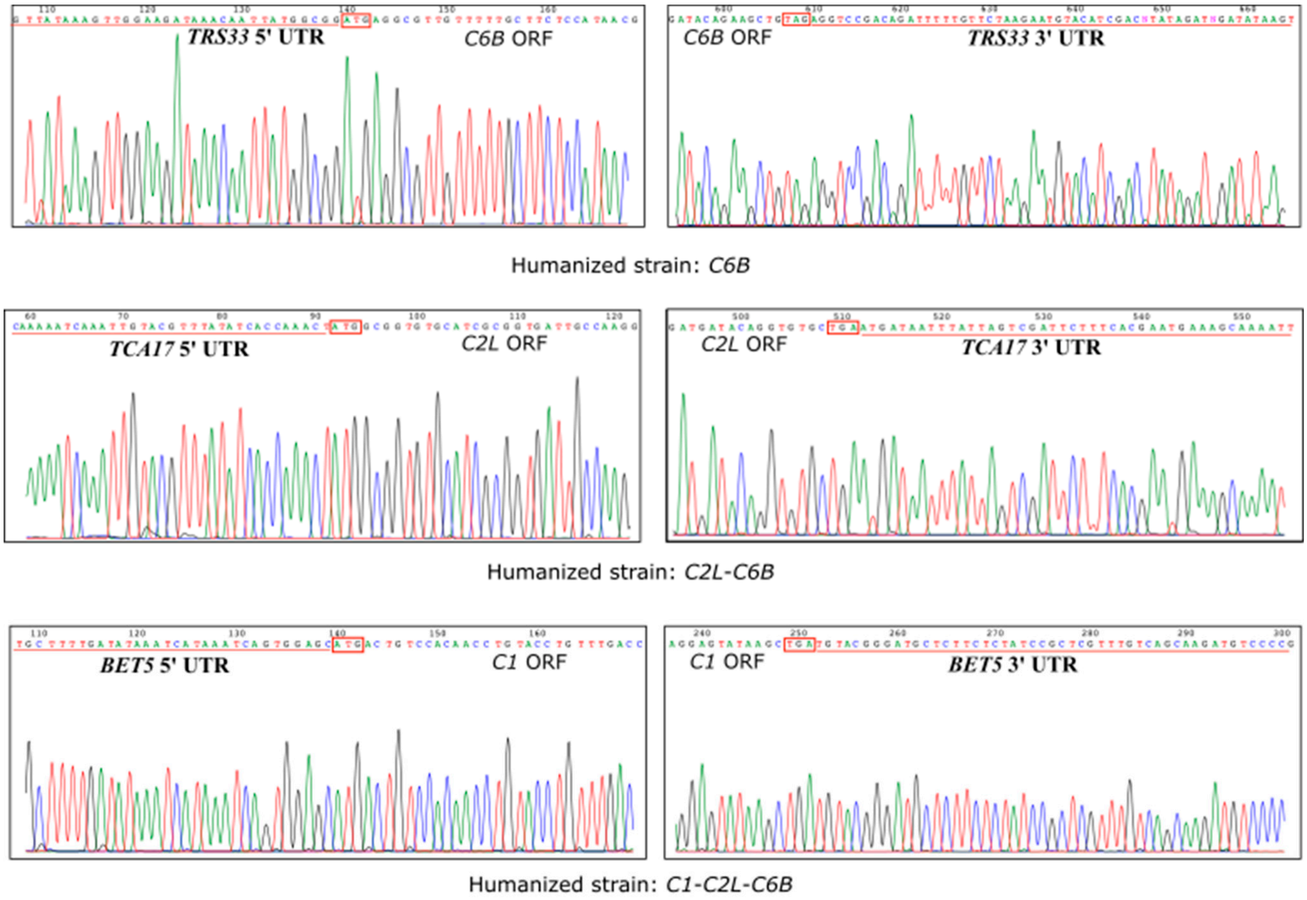

Figure S1. Sanger sequencing of C1, C2L, and C6B. Genomic DNA from each humanized yeast strain was PCR-amplified and subjected to Sanger sequencing. The red boxes indicate the start and stop codons of the integrated human open reading frames (ORFs), while the underlined regions correspond to the upstream and downstream untranslated regions (UTRs) of the endogenous yeast genes. These chromatograms confirm scarless replacement of the yeast ORFs with their human counterparts, validating precise genomic integration for each construct.

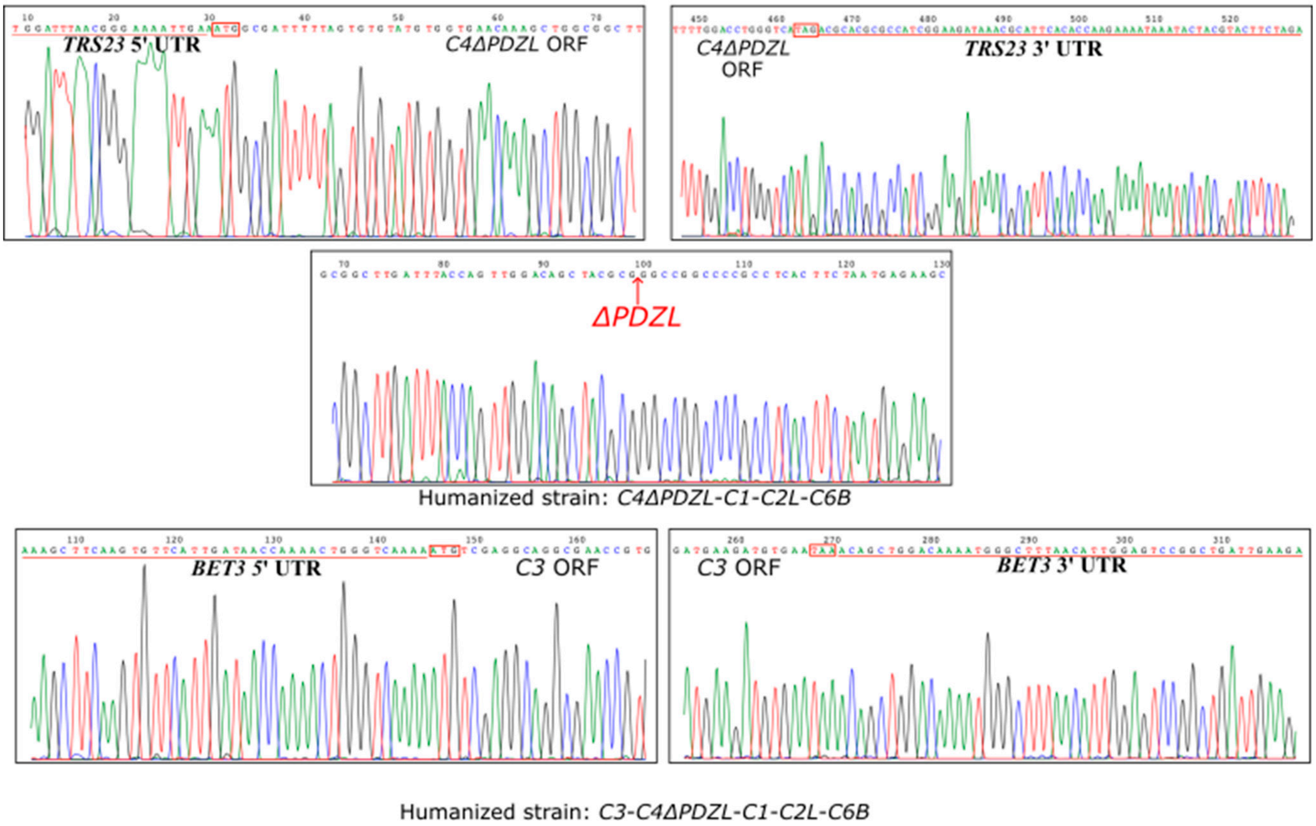

Figure S2. Verification of successful C4ΔPDZL and C3 scarless replacement via Sanger sequencing. Chromatograms from Sanger sequencing confirm the seamless integration of human C4ΔPDZL and C3 ORFs into the yeast genome. The sequences show the expected junctions between yeast 5′ and 3′ untranslated regions (UTRs) and the inserted human ORFs, demonstrating scarless replacement. For C4ΔPDZL, the characteristic ΔPDZL region is indicated.

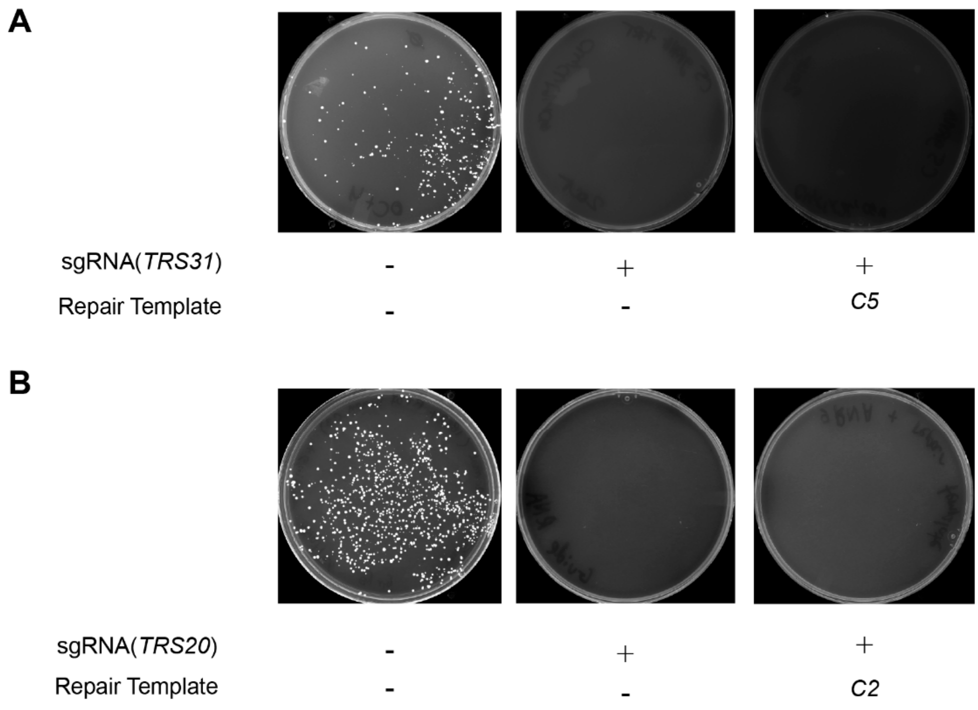

Figure S3. CRISPR-Cas9-mediated humanization of C5 and C2 in a PHTC background. All transformations were performed in the PHTC strain. The top panel shows targeting of the TRS31 gene using a sgRNA and a repair template encoding C5. The bottom panel shows targeting of the TRS20 gene using a sgRNA and a repair template encoding C2. No colonies are observed in the plate that contains the sgRNA with the repair template, indicating unsuccessful replacement.

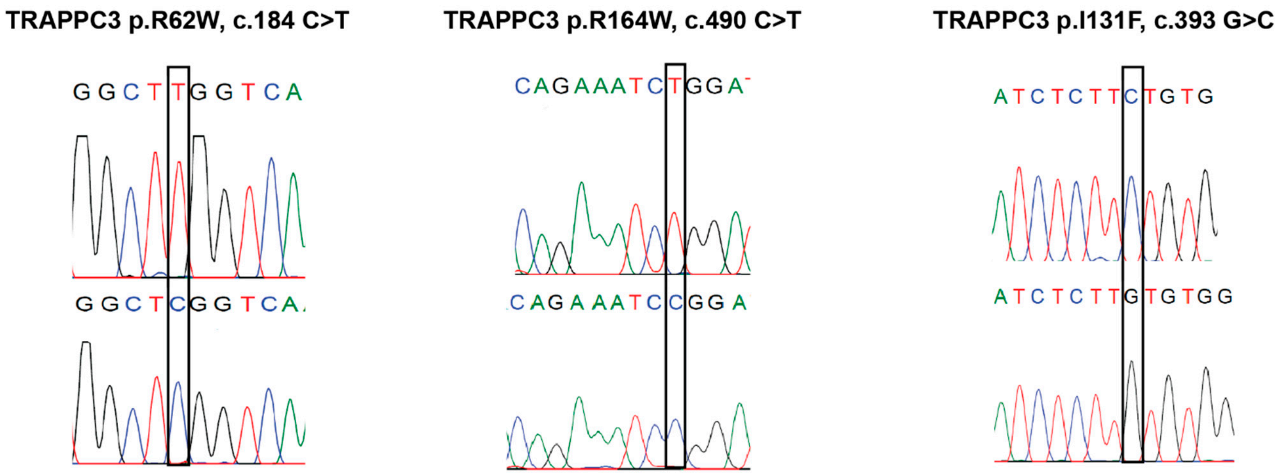

Figure S4. Sanger sequencing confirmation of *C3* variants in the humanized yeast strain The chromatograms show successful integration of the indicated *C3* variant ORFs. The boxed base indicates the nucleotide change introduced by CRISPR-Cas9-mediated genome editing.
